# Local ligand concentration gradients induced by the plasma membrane

**DOI:** 10.1016/j.isci.2025.112954

**Published:** 2025-06-19

**Authors:** Ágnes Szabó, Gabriella Tóth, Tímea Szatmári, Gábor Mocsár, István Rebenku, János Szöllősi, Peter Nagy

**Affiliations:** 1 Department of Biophysics and Cell Biology, Faculty of Medicine, University of Debrecen, Debrecen, Hungary; 2 HUN-REN-UD Cell Biology and Signaling Research Group, Faculty of Medicine, University of Debrecen, Debrecen, Hungary

**Keywords:** theoretical physics, biological sciences, membranes, biophysics, computer simulation

## Abstract

The plasma membrane is a dynamic structure surrounded by the extracellular matrix. Although stimulation of transmembrane proteins by soluble ligands takes place in this environment, its influence on receptor activation is usually overlooked. In the current manuscript, we quantitatively measured the concentration of epidermal growth factor (EGF) at the cell membrane and found that ligand distribution is non-homogeneous due to two concentration peaks causing significantly higher EGF concentrations at the membrane than in the bulk solution. Using experiments, calculations, and simulations, we show that the membrane-proximal peak is caused by membrane turnover, while the more distant one, only present in ErbB2-expressing cells, is generated by locally hindered diffusion possibly caused by the extracellular matrix. Both theory and experiments reveal that these phenomena increase the apparent affinity and decrease the apparent cooperativity of the receptor-ligand complex. Interpretation of the effects of soluble ligands interacting with receptors must involve this non-homogenous concentration profile.

## Highlights

• Concentration of externally applied ligands near the cell membrane is heterogeneous
• The membrane proximal peak is related to membrane turnover
• The membrane distal peak is related to increased microviscosity
• This phenomenon may alter the apparent affinity and cooperativity of receptors

## Graphical abstract

**Figure:**
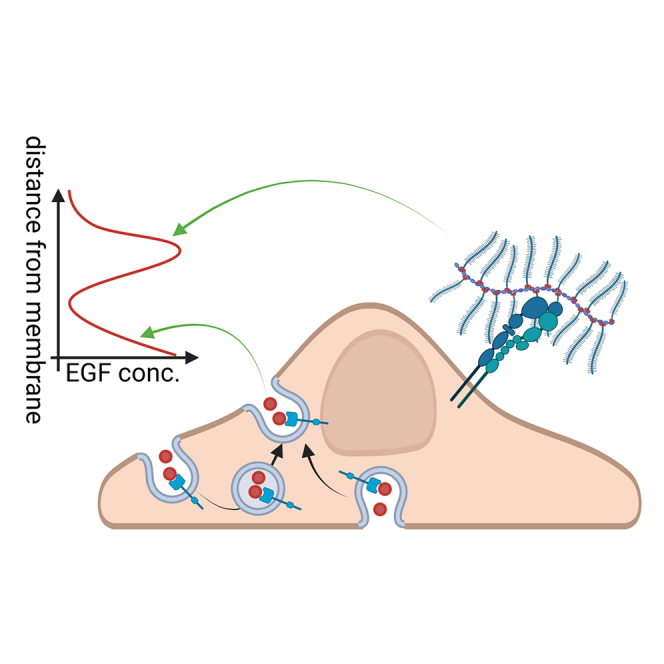

## Introduction

The plasma membrane not only presents a controlled, semipermeable barrier between the cell’s interior and the outside world, but it is also home to membrane proteins constituting ∼25% of the human proteome and ∼60% of current drug targets.^1^ Although transmembrane signaling is initiated by ligand binding to membrane receptors, the interest of conventional research is focused on events, such as phosphorylation, taking place later, on the intracellular side of the membrane. Quantitative biophysical methods are beginning to reveal the complexities of ligand-induced and constitutive receptor oligomerization that is regulated by protein-protein interactions, membrane domains, and the cytoskeleton.^2^ As a result, the concept of the activation of the epidermal growth factor receptor (EGFR) has transitioned from a simple, ligand-induced dimerization model to a complex picture involving a dynamic equilibrium between different dimeric and oligomeric species.^3^^,^^4^ Apart from ligand-induced clusters, constitutive, ligand-independent dimers and oligomers have been shown to exist.^5^^,^^6^^,^^7^^,^^8^ Although the requirement for ligand-enhanced dimerization of EGFR monomers still remains the cornerstone of models of receptor activation,^3^ organization of inactive receptors into constitutive, large-scale complexes and their rearrangement into ligand-activated oligomers account for certain aspects of autoinhibition and super-stoichiometric signaling at ultralow ligand concentrations.^9^^,^^10^ Furthermore, EGFR is only one of the members of the ErbB family of receptor tyrosine kinases, and other members of ErbB proteins (ErbB2-4) can form heterodimeric interactions with EGFR, also known as ErbB1.^11^ In particular, ErbB2, a ligandless co-receptor of proper, ligand-binding ErbB receptors, increases the signaling potency and ligand binding affinity of EGFR through heterodimerization although ErbB2 itself does not bind the growth factor directly.^12^ However, ligand binding itself may also result in remarkable complexity due to the affinity of receptors depending on ligand type^13^ and the ligand-bound state of the receptor dimer leading to cooperativity effects.^14^ Growth factor binding must also be influenced by the convoluted and dynamic nature of the plasma membrane.^15^ This phenomenon has been largely neglected as even quantitative studies are usually based on the simplistic assumption of a gradientless ocean of ligand leading to a homogeneous concentration in the extracellular space. Deviations from models assuming an inexhaustible reservoir of ligand invalidate the application of the principle of deterministic mass action in a well-mixed environment. Several instances of such unexpected phenomena have been described, such as apparent increase in receptor affinity due to clustering and hindered diffusion facilitating rebinding of dissociated ligands.^16^^,^^17^^,^^18^ Furthermore, preassembled clusters of G-protein-coupled receptors were found to respond to ultralow, femtomolar ligand concentrations, which is lower by several orders of magnitude than the threshold predicted by simple mass-action principles.^19^ Such ultralow ligand concentrations require the application of stochastic reaction kinetics due to the large relative fluctuation of local concentrations.^20^^,^^21^ The universal nature of homogeneous ligand distributions has been explicitly questioned by observing ligand gradients. Several biologically relevant mechanisms can lead to such a phenomenon. (1) A membrane-soluble β-adrenoreceptor blocker was found to be present at a concentration at the plasma membrane that was by more than one order of magnitude higher than in the bulk solution,^22^ a phenomenon that was attributed to the ligand partitioning in the membrane.^23^^,^^24^ (2) Spatially modulated diffusion-state switching has also been predicted and found to lead to concentration gradients.^25^^,^^26^ (3) Morphogen gradients are widely known in developmental biology, and these stable concentration gradients are attributed to continuous flow generated by localized production of ligands.^27^ (4) Theory predicts that transient gradients of intracellular messengers, activated membrane proteins, and extracellular ligands are generated due to membrane curvature and turnover,^15^ and the observation of transient accumulation of activated bradykinin receptors at regions of higher membrane curvature supports these conclusions.^28^

In the current manuscript, we identified two different kinds of EGF gradients in the vicinity of the plasma membrane. Calculations and experimental evidence show that these ligand gradients are due to membrane turnover and local viscosity gradients. Such phenomena must not be overlooked in quantitative studies of ligand action.

## Results

### EGF concentration in the vicinity of the plasma membrane is not homogeneous

**Figure 1:**
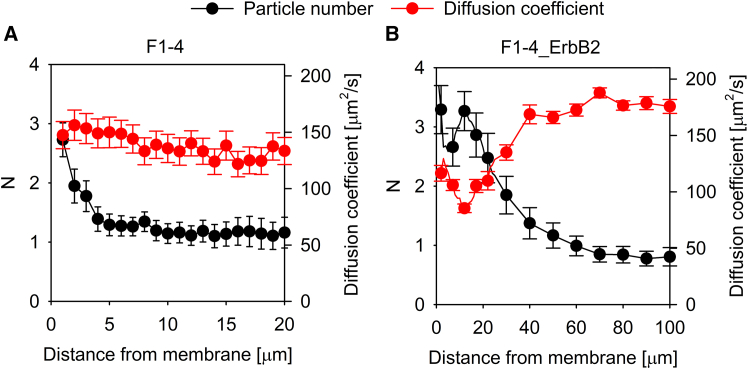
EGF concentration gradients in the vicinity of the plasma membrane (A and B) EGFR-expressing F1-4 (A) and EGFR-ErbB2-coexpressing F1-4_ErbB2 cells (B) were incubated in the presence of 10 nM TAMRA-EGF. EGFR and ErbB2 expressions by the transfected cells were identified based on the GFP and CFP fluorescence, respectively, of the corresponding construct. Point FCS measurements were performed every μm up to 20 μm from the membrane, then every 10 μm up to a distance of 100 μm from the plasma membrane. The number of particles per confocal volume (*N*) and the diffusion coefficient were determined by fitting. Due to the absence of any feature at large distances from the membrane, curves for F1-4 cells are only displayed up to 20 μm. The error bars represent the standard deviation.

Scientists performing cell biological experiments take it for granted that the bulk ligand concentration is equal to that in the immediate vicinity of plasma membrane receptors, especially in the case of hydrophilic ligands whose partitioning in the lipid bilayer does not take place. Given the dynamic and convoluted nature of the plasma membrane, we set out to investigate this tenet for EGF whose potentially different concentration at the plasma membrane may have important repercussions for interpreting the affinity and cooperativity of receptor-ligand interactions. Two cell lines were used throughout the experiments. Both of them were generated from CHO cells devoid of endogenous expression of ErbB proteins.^29^ F1-4, stably transfected to express GFP-tagged EGFR,^30^ was used to characterize EGFR-specific effects without the influence of other ErbB proteins, while F1-4_ErbB2 cells, generated by transiently transfecting F1-4 with CFP-tagged ErbB2, were used for measuring the possible effect of ErbB2 coexpression. Ligand concentrations as a function of distance from the plasma membrane were determined by fluorescence correlation spectroscopy (FCS). F1-4 cells were incubated in the presence of 10 nM fluorescently labeled EGF, and the concentration of the peptide growth factor was measured up to 100 μm above the plasma membrane. The concentration of EGF exhibited a membrane-proximal peak extending approximately 5 μm from the membrane, with a peak concentration reaching 3-times the bulk EGF concentration (Figure 1A). While this membrane-proximal peak was also present in F1-4_ErbB2 cells, these cells also displayed a second, more distal EGF peak with a magnitude similar to the membrane-proximal one with a peak at ∼10–20 μm from the membrane (Figure 1B). In both cell lines, EGF concentrations far from the plasma membrane leveled off at ∼1 particle/confocal volume, which corresponds to the expected bulk concentration of 10 nM according to Equation 3. When the EGF concentration was measured above cell-free areas of the coverslip, no ligand concentration gradients were detected implying that the observed inhomogeneous distribution is attributable to cells (Figure S2A). When the same experiments were carried out with formaldehyde-fixed cells, the membrane-proximal peak was absent for both cell lines, but the membrane-distal EGF concentration peak was present in F1-4_ErbB2 cells (Figures S2B and S2C). We concluded that EGF concentrations in the vicinity of the plasma membrane substantially deviate from homogeneity, and some of these phenomena are due to the presence of live cells.

### The membrane-distal peak of EGF concentration is caused by locally hindered diffusion

**Figure 2:**
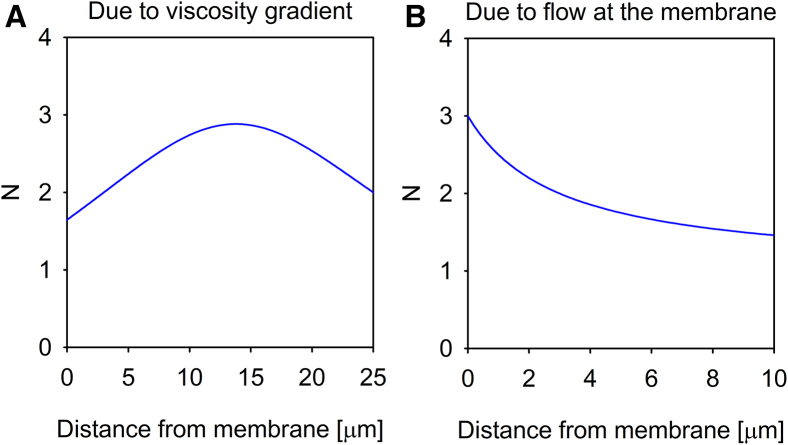
Simulation of ligand concentration gradients due to viscosity gradients and flow (A) Local heterogeneity in the diffusion coefficient of TAMRA-EGF determined by FCS in F1-4_ErbB2 cells was modeled analytically (Figure S3), followed by solving the Fokker-Planck equation for steady state. The concentration gradient of TAMRA-EGF generated as a result of the local minimum of the diffusion coefficient (“Due to viscosity gradient”) is shown in (A). Details of the calculation are described in section “calculation of the local concentration gradient due to locally hindered diffusion” of STAR Methods. (B) Fick’s second law of diffusion was solved with boundary conditions corresponding to the measured TAMRA-EGF concentrations for F1-4 cells (*N* = 3 at the membrane and *N* = 1 at infinite distance from the membrane). The shape of the concentration profile is shown in (B). Details of the calculation and the determination of the required flow to maintain the concentration gradient are described in section “calculation of the concentration gradient modified by flow” of STAR Methods.

**Figure 3:**
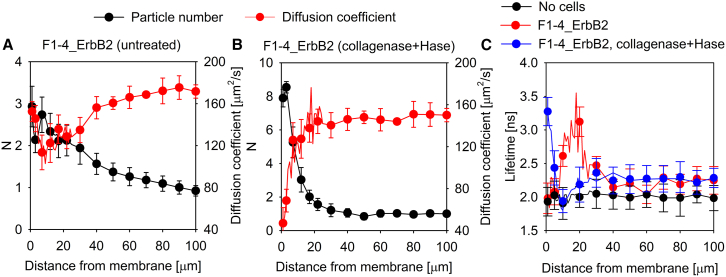
The role of the extracellular matrix and local microviscosity changes in the generation of the membrane distal EGF concentration peak (A and B) F1-4_ErbB2 cells expressing both EGFR and ErbB2 were incubated with 10 nM TAMRA-EGF (A). Another sample of cells was pretreated with 20 μg/mL collagenase and 20 U/mL hyaluronidase at 37°C for 90 min before the addition of 10 nM TAMRA-EGF (B). The number of ligand particles per confocal volume (*N*) and the diffusion coefficient of TAMRA-EGF were determined by FCS at every micrometer up to 20 μm from the membrane, and then at every 10 μm up to 100 μm above the plasma membrane. (C) The fluorescence lifetime of a molecular rotor, 8-Phenyl-BODIPY, reporting on the local microviscosity was measured in the medium in the absence of cells, above F1-4_ErbB2 cells and above F1-4_ErbB2 cells treated with 20 μg/mL collagenase and 20 U/mL hyaluronidase. The error bars represent the standard deviation.

One of the possible mechanisms capable of generating a stable concentration gradient is spatially varying diffusion coefficient due to e.g., a local viscosity gradient. Since flux is proportional to the product of the diffusion coefficient and the concentration, equilibrium in the presence of spatially varying diffusivity can only be maintained if the concentration also exhibits spatial variation with concentration peaks coinciding with troughs in diffusivity. Using FCS, we also measured the diffusion coefficient and plotted its correlation with the local concentration of EGF. While there was no gradient in the diffusion coefficient around F1-4 cells (Figure 1A), the membrane distal concentration peak coincided with a local minimum of the diffusion coefficient for F1-4_ErbB2 cells (Figure 1B). While the lack of correlation between diffusivity and concentration rules out spatially varying diffusion coefficient as an explanation for the membrane proximal peak, the membrane distal EGF concentration peak present only in F1-4_ErbB2 cells may be caused by locally hindered diffusion. In order to find out if the extent of the local minimum in the diffusion coefficient is sufficient to account for the membrane distal EGF concentration peak, we solved the Fokker-Planck equation using the experimentally determined diffusion coefficients to obtain the concentration gradient due to the locally hindered diffusion. Details of the calculation are described in section “calculation of the local concentration gradient due to locally hindered diffusion” in STAR Methods. The magnitude of the concentration gradient generated by the local minimum in diffusivity is 1–1.5 particles/confocal volume, similar to the experimentally obtained concentration gradient observed in the case of the membrane-distal peak in F1-4_ErbB2 cells (Figure 2A). These results show that the local minimum in the diffusion coefficient at ∼10–20 μm from the membrane of F1-4_ErbB2 cells is sufficient to account for the spatially coinciding peak in the concentration of EGF. An obvious explanation for the local minimum in diffusivity is a local maximum of viscosity due to secretion of extracellular matrix components. Therefore, we measured the correlation between the diffusion coefficient and the EGF concentration in F1-4_ErbB2 cells treated with a mixture of collagenase and hyaluronidase. While the treatment made the membrane distal peak disappear, it resulted in a very strong ligand concentration peak right above the membrane (Figure 3). This large concentration peak coincided with a very marked decrease in the diffusion coefficient indicating that the former may be caused by the latter. We attribute this observation to degradation of the extracellular matrix by the enzymatic treatment and deposition of its fragments on the plasma membrane.

Imaging of the extracellular matrix around the membrane-distal EGF concentration peak was not successful, likely due to the fact that a low number of obstacles can lead to significantly retarded diffusion, and to the unknown composition of the matrix secreted by Chinese hamster ovary cells.^31^^,^^32^ Therefore, we performed additional FCS experiments to show that the membrane-distal concentration peak and the corresponding local minimum in the diffusion coefficient is not peculiar to EGF. To this end, the concentration and diffusion coefficient of TAMRA-labeled bovine serum albumin and free TAMRA were measured, and both molecules displayed a concentration peak and a coinciding minimum in the diffusion coefficient at ∼20 μm from the plasma membrane, just like TAMRA-EGF, without any sign of the membrane proximal peak (Figure S4). These experimental results imply that the membrane proximal peak has to do with processes specific to EGF, but the membrane distal one is not specific to a certain molecule type or molecular weight. Furthermore, we applied a BODIPY-based molecular rotor whose fluorescence lifetime reports on the local microviscosity.^33^^,^^34^ In the absence of cells, the lifetime of the sensor was constant in the solution, whereas it displayed a maximum around 10–20 μm above the membrane of F1-4_ErbB2 cells indicating a local increase in microviscosity (Figure 3C). This region of high viscosity moved to the membrane in cells treated with a combination of collagenase and hyaluronidase (Figure 3C) just as the local minimum of the diffusion coefficient was just above the membrane in the cells treated with the digestive enzymes (Figure 3B). It can be concluded that the membrane distal peak 10–20 μm from the membrane of F1-4_ErbB2 cells is caused by a local maximum of viscosity, which is likely due to the presence of extracellular matrix components.

### The membrane-proximal peak in EGF concentration is related to membrane turnover

Abolishment of the membrane proximal EGF concentration peak in formaldehyde-fixed cells clearly showed that live cells are required for the presence of this peak (Figures S2B and S2C). Furthermore, the lack of correlation between the diffusion coefficient and the EGF concentration rules out local viscosity as a potential cause for the membrane proximal peak (Figure 1). Therefore, we considered binding to membrane receptors as a potential cause for the membrane proximal EGF concentration peak as specific or non-specific binding of ligands to the plasma membrane have been suggested to account for higher local ligand concentrations at the cell membrane.^22^ We simulated the establishment of an equilibrium EGF concentration profile taking receptor binding and diffusion of EGF into consideration. The simulations clearly show that no ligand concentration gradient is present upon reaching equilibrium, essentially ruling out binding as the cause for the membrane proximal peak (Figures S5A and S5B). The calculations are described in detail in “simulation of the concentration gradient generated by binding and exocytosis” in STAR Methods.

**Figure 4:**
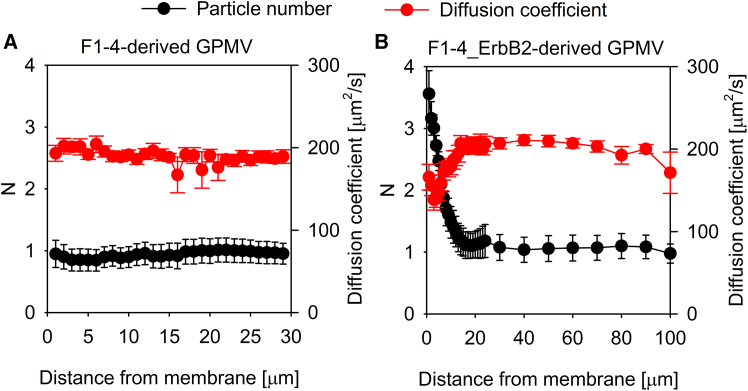
The concentration gradient of TAMRA-EGF in the vicinity of GPMVs (A and B) GPMVs were prepared from F1-4 (A) and F1-4_ErbB2 cells (B) and they were seeded on poly-L-lysine-coated coverslips. The vesicles were incubated with 10 nM TAMRA-EGF and the number of particles per confocal volume (*N*) and the diffusion coefficient of TAMRA-EGF were determined by FCS. The error bars represent the standard deviation.

We assumed that active membrane turnover may be behind the generation of the EGF concentration peak in the immediate vicinity of the plasma membrane. Giant plasma membrane vesicles (GPMVs) are useful tools for elucidating the contribution of membrane trafficking to biological phenomena since their composition largely resembles that of the plasma membrane, but they do not undergo membrane turnover.^35^ While the ligand concentration around GPMVs generated from F1-4 cells was completely homogeneous, it showed a membrane proximal peak in the case of F1-4_ErbB2 cells (Figure 4). Since this concentration peak was inversely correlated with the diffusion coefficient (Figure 4B), it must be generated by locally hindered diffusion. Together with the results obtained with formaldehyde-fixed cells, these experiments imply that a living cell membrane displaying active turnover is required for the generation of the membrane proximal EGF concentration peak.

Given that specific binding to receptors or non-specific binding to a reservoir cannot account for the permanent, positive concentration peak at the membrane (Figures S5A and S5B), we performed simulations to see what kind of effects the appearance of ligand-saturated EGF receptor or free ligand have on the local EGF concentration at the membrane. Transient appearance of liganded EGF receptor results in a transient, positive concentration peak (Figure S5C), while continuous appearance of EGF (Figure S5D) leads to a permanent, membrane-proximal concentration peak. A similar result was obtained by continuously adding liganded EGF receptor to the plasma membrane (data not shown). These simulations imply that a ligand source at the plasma membrane is able to generate a local concentration peak.

In order to delve deeper into the cause of the membrane-proximal ligand concentration peak, we attempted to inhibit membrane turnover without compromising cell viability. In accordance with previous findings, individual inhibitors were not sufficient to achieve sufficient inhibition of membrane turnover.^36^ Finally, we used a three-component cocktail containing latrunculin B, para-amino blebbistatin, and myristoylated dynamin inhibitory peptide (DIP) blocking actin polymerization, myosin, and dynamin activity, respectively. Treatment of F1-4 cells with this combination of inhibitors abolished the membrane proximal peak but replaced it with another one (Figures S6 and 1A). The inverse correlation of this new concentration peak with the diffusion coefficient (Figure S6) implies the development of a diffusion barrier whose possible origin will be considered in the discussion.

**Figure 5:**
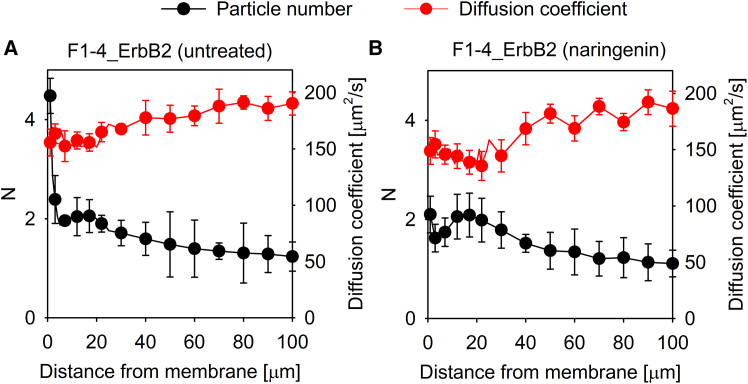
The effect of blocking TPC2 two-pore ion channels on the development of ligand gradients in the vicinity of the plasma membrane (A and B) F1-4_ErbB2 cells (A) and naringenin-pretreated F1-4_ErbB2 cells (B) were incubated in the presence of 10 nM TAMRA-EGF followed by the determination of the number of ligands per confocal volume (*N*) and the diffusion coefficient of TAMRA-EGF by FCS. The error bars represent the standard deviation.

The previous results implicate membrane turnover in the generation of the membrane-proximal EGF concentration peak. Although this process must involve exocytosis, a widely used inhibitor of exocytosis of Golgi-derived and recycling vesicles^37^^,^^38^^,^^39^ was unable to abolish the membrane-proximal ligand concentration peak (Figure S7). A possible way how membrane turnover, i.e., very fast succession of endocytosis and exocytosis, may lead to a membrane proximal ligand concentration peak is the immediate re-exocytosis of endocytosed content after it has been concentrated due to shrinkage of endosomes. Two-pore ion channels have been implicated to take part in this process.^40^^,^^41^ Treatment of F1-4_ErbB2 cells with naringenin, a recently described inhibitor of two-pore ion channels,^42^ significantly reduced the magnitude of the membrane proximal peak without an effect on the membrane-distal one (Figure 5). It is worth pointing out that the shape of the membrane-distal peak in these experiments is somewhat different from those shown in Figure 1. We assume that the extracellular diffusion barrier built by the cells was somewhat different in these experiments from that shown in Figure 1 resulting in a bit less pronounced membrane-distal peak. Given the complexity of the phenomena leading to these observations, we believe that these differences are well within the acceptable range of biological variability.

The previous lines of evidence suggest that membrane turnover involving flicker fusion^43^ may be the cause for the appearance of the membrane proximal EGF concentration peak. The frequency and speed of this process mandate that the reservoir of the ligand released as part of membrane turnover be very close to the plasma membrane. Brief treatment of cells with an acidic solution (pH 2.8) completely removed ligands bound to plasma membrane receptors of cells incubated with the ligand on ice, i.e., from those binding sites around which the pH is acidified. However, when cells were incubated with fluorescent EGF at room temperature, acid treatment only partially reduced their fluorescence (Figure S8). Remarkably, a part of the remaining EGF fluorescence appeared to be “membrane-like” implying that a significant fraction of internalized EGF is practically within a couple of hundred nanometers from the plasma membrane. In summary, simulations, experiments with inhibitors and GPMVs as well as imaging of the distribution of fluorescent EGF strongly suggest that rapid membrane turnover is the likely cause of the development of the membrane proximal EGF concentration peak.

### Potential consequences of local, membrane-proximal concentration gradients on experimental results

**Figure 6:**
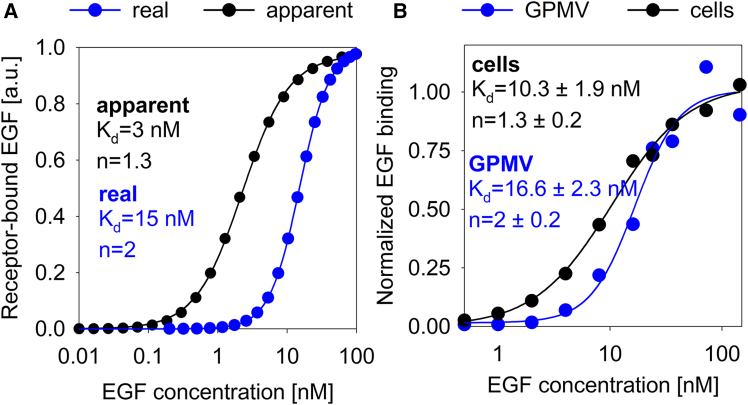
The effect of the membrane-proximal ligand concentration gradient on the apparent cooperativity of ligand binding (A) An EGF concentration series ranging from 0.01 to 100 nM was simulated. Due to the EGF concentration-dependent, membrane-proximal ligand concentration peak, the actual EGF concentration at the membrane is different from the bulk concentration. An approximate relationship between the assumed and real concentration of the ligand at the membrane was established based on experimental data (Figure S9), and the actual ligand concentrations at the membrane were calculated. The concentration of receptor-bound EGF was calculated using these real EGF concentrations and the Hill equation assuming a dissociation coefficient of 15 nM and a Hill coefficient of 2 (blue symbols). The data points were fitted by the Hill equation, which faithfully reproduced both the dissociation constant and the Hill coefficient (blue line). In an experiment, one would assume that the EGF concentration at the membrane is equal to the bulk ligand concentration, and an apparent relationship would be established between the bulk ligand concentration and the measured, receptor-bound EGF concentration (black symbols). The Hill equation was fitted to this dataset providing an apparent dissociation constant of 3 nM and an apparent Hill coefficient of 1.3 (black line). (B) F1-4 cells (black) and F1-4-derived GPMVs (blue) were incubated with a concentration series of TAMRA-EGF, and the membrane was identified based on the GFP-fluorescence of the EGFR-GFP construct in confocal microscopic images (Figure S11). The intensity of membrane-bound TAMRA-EGF was determined and normalized to the GFP fluorescence and then to the maximum TAMRA intensity observed at the highest ligand concentration. The Hill equation was fitted to the experimental datasets producing the dissociation and Hill coefficients displayed in the figure.

The previous experimental results unequivocally show that the local ligand concentration at the plasma membrane is different from the bulk concentration, i.e., the concentration the experimenter believes to exist at the membrane. We argued that the fold-increase in the local ligand concentration at the membrane may depend on the bulk concentration if it is indeed the result of active membrane turnover. Given the complexity of the experiments, we only measured the EGF concentration profile as a function of distance from the membrane at three bulk ligand concentrations (1, 10, and 100 nM). While we observed a ∼3-fold increase in the membrane proximal EGF concentration at 10 nM bulk EGF concentration (the concentration used throughout the manuscript), the membrane proximal EGF concentration peak was practically absent at 100 nM bulk ligand concentration. On the contrary, the fold-increase in the local EGF concentration was even more pronounced at 1 nM bulk EGF concentration (Figure S9). The fact that the extent of the membrane effect depends on the bulk EGF concentration has a sinister implication since experimentally observed concentration dependencies may get distorted. The cooperativity and affinity of ligand binding to the EGF receptor have been the subject of intense research for decades.^14^ In order to see how experimentally determined affinity and cooperativity may be influenced by the concentration dependence of the membrane proximal ligand concentration peak, we applied a power function to model the clearly nonlinear relationship between the membrane-proximal EGF concentration and the bulk EGF concentration (Figure S10). Then, we simulated an equilibrium, ligand binding experiment. An *in-silico* EGF concentration gradient was generated corresponding to the experimentally applied, bulk EGF concentrations. Using the relationship established in Figure S10, the EGF concentrations at the membrane were calculated followed by determining the amount of receptor-bound EGF assuming a single binding site characterized by an affinity of 15 nM and a Hill coefficient of 2 (Figure 6A). In an experimental setting, the experimenter would fit the Hill equation on the plot of these observed, receptor-bound EGF concentrations as a function of the bulk EGF concentrations. This fitting resulted in a higher apparent affinity and a lower apparent cooperativity (Figure 6A).

Finally, we wanted to check whether this predicted effect is observed experimentally. We measured the equilibrium binding of fluorescent EGF to F1-4 cells (in which the membrane proximal ligand concentration peak is present) and in F1-4-derived GPMVs devoid of the membrane proximal EGF concentration peak (Figure S11). Fitting the Hill equation to the experimental data of cells resulted in higher affinity and lower cooperativity than when the data of GPMVs was evaluated (Figure 6B). Since the bulk EGF concentration is equal to the membrane-proximal concentration for GPMVs, these fitted parameters correspond to the real values. The fitted parameters obtained with cells are distorted by the membrane proximal EGF concentration peak and therefore correspond to the apparent concentration dependence in the simulated experiment. Even though GPMVs and living cells obviously differ in ways other than the membrane-proximal EGF concentration peak, the fact that the difference between the affinity and cooperativity of EGF binding to cells and GPMVs differ as predicted shows that the presence of local ligand concentration gradients may significantly derail the interpretation of experimental results.

## Discussion

We have witnessed a transition in our views regarding the plasma membrane from a static barrier to a system that is dynamic in both space and time.^15^^,^^44^ In the present manuscript, we not only present convincing evidence for how this dynamism alters ligand concentrations in the vicinity of the cell membrane but also hint at possible consequences of this phenomenon. When trying to put these results into the context of cell biological phenomena, let us first consider what kind of processes can generate stable concentration gradients. Physical principles and previous experimental evidence show that concentration gradients are either generated by (1) local alterations in diffusivity (i.e., spatial changes in the diffusion coefficient) or by (2) continuous, local production of ligands, a well-known phenomenon for establishing concentration gradients of morphogens.^27^^,^^45^ Diffusion, which dissipates gradients, is obviously unable to generate gradients on its own. Local alteration of diffusivity is a spatially and temporally stable pattern of the diffusion coefficient, which is capable of generating a gradient (see section “calculation of the local concentration gradient due to locally hindered diffusion” in STAR Methods). A temporally and spatially stable pattern of the diffusion coefficient can be brought about by (1) switching, spatially modulated conformational changes^25^^,^^26^; (2) local viscosity gradients; or by (3) size-dependent changes in the diffusion constant near membrane surfaces,^46^ all of which have been shown to give rise to concentration gradients. We can rule out the last mechanism since this effect was shown to be significant only for molecules much larger than EGF and it was only observable on the nanometer scale, whereas the concentration gradients reported here are detected micrometers from the plasma membrane. While not generating concentration gradients or changes in the diffusion coefficient, the proximity of a fluctuating membrane can bring about an artifact resulting in changes in the autocorrelation function.^47^ We can rule out the contribution of this effect to the concentrations calculated from the autocorrelation functions since the fluctuating appearance of the membrane in the confocal volume would have resulted in a deviation of the autocorrelation function from the equation describing single-component, 3D diffusion, which was not the case (see Figure S1). Switching, spatially regulated conformational changes are also unlikely to play a role since conformational changes of EGF affecting its diffusion coefficient are unknown. As far as the membrane-proximal EGF concentration peak is concerned, local viscosity gradients can also be ruled out since this EGF gradient was uncorrelated with the diffusion constant. This reasoning leaves the local source (local production) as the only possible explanation for the membrane-proximal EGF concentration peak. It is important to point out that reversible, equilibrium binding to membrane receptors or to the lipid bilayer cannot cause a stable concentration gradient (shown by simulations in Figure S5) since a concentration gradient, in the absence of a change of diffusivity, generates flow, which depletes the concentration peak. A constant source is therefore needed to replenish the concentration peak depleted by flow.

From a strictly biophysical point of view, what can we say about the cause of the membrane-proximal ligand concentration gradient? (1) It cannot be produced passively since spontaneous buildup of a chemical potential gradient would be a violation of the 2^nd^ law of thermodynamics; therefore (2) it must be some kind of active biological process (also supported by the fact that it is absent from formaldehyde-fixed cells and from GPMVs); but (3) it must be a process leading to a biological equilibrium since the concentration gradient is temporally stable.

What kind of biological process is capable of producing the temporally stable, membrane proximal ligand concentration peak? Treatment of cells with an inhibitor cocktail blocking actin polymerization, myosin and dynamin activity, and consequently membrane turnover, abolished the membrane-proximal ligand concentration peak, but replaced it with another one. Although this experimental result implies that membrane turnover is involved in the generation of the membrane-proximal ligand concentration peak, the appearance of a new peak calls for an explanation. Why do we believe that these two ligand concentration peaks are of different kinds? In untreated F1-4 cells, the peak is within 5 μm of the plasma membrane and it is uncorrelated with the diffusion coefficient, while it extends up to ∼10 μm in the cocktail-treated cells and it is inversely correlated with the diffusion coefficient. This latter observation can be rationalized by the exocytosis-triggering effect of latrunculin B, resulting in the fast release of secretory vesicles.^48^^,^^49^ While we are uncertain about the contents of the vesicles whose release is induced by latrunculin B, the inverse correlation between the diffusion coefficient and the number of particles clearly implies that a diffusion barrier has been generated in the extracellular space.

In search of the possible biological mechanism for the generation of the membrane-proximal ligand concentration peak, we showed by simulations that a continuous ligand source at the membrane will generate an EGF concentration peak (Figure S5). According to our calculations, ∼600 EGF molecules have to appear in a second in 1 μm^2^ membrane area to generate the experimentally observed concentration gradient. Details of the calculations are described in section “calculation of the concentration gradient modified by flow” in STAR Methods. To the best of our knowledge, EGF is not produced by CHO cells, but even if it were, the release of 600 EGF molecules every second in a square micrometer membrane area would result in the release of 3.6 × 10^13^ EGF molecules in 10 min (assuming there are 10^5^ cells in a well each of them having a surface area of 1,000 μm^2^; 600 EGF/s/μm^2^ ⋅ 1,000 μm^2^ ⋅ 600 s ⋅ 10^5^ = 3.6 × 10^13^) equivalent to 6 × 10^−11^ mol, which corresponds to a concentration of 600 nM assuming an incubation volume of 100 μL. Since there is no hint at such gross alterations in the bulk EGF concentration, production of EGF by secretion or cleavage is not a viable explanation. In order for a constant gradient to be maintained, it has to be assumed that membrane turnover, that is, re-exocytosis of endocytosed material is behind the membrane-proximal EGF peak. Our simulations showed that the appearance of both liganded receptors and free ligand can produce the source required for the maintenance of the concentration gradient (Figure S5). Assuming the radius of an endocytic vesicle is between 0.1 and 0.2 μm and the ligand concentration is 10 nM, the number of free EGF molecules in a vesicle ranges between 0.02 and 0.2. According to reasonable estimates, the number of internalized, liganded EGF receptors ranges between a few to 20 per vesicle. Therefore, we assumed that the total number (liganded + free) of EGF molecules in a vesicle is on the order of 10. Consequently, the number of vesicles endocytosed and re-exocytosed must be in the range of ∼60 vesicles per square micrometer per second (600 EGF/μm^2^/s / 10 EGF/vesicle). For synapses, this magnitude of vesicle traffic is well within the accepted range.^50^ For non-neural cells, the exact numbers are usually unknown, but there are a number of active and fast membrane turnover mechanisms that can contribute to such high vesicle traffic.^51^ Given the lack of effect of monensin, an inhibitor of conventional exocytic pathways,^37^^,^^38^^,^^39^ the membrane-proximal EGF concentration peak must be caused by membrane dynamism and turnover, variably termed kiss-and-run, open and closed, or flicker exocytosis, in which proper vesicles are not formed or exocytosed in their entirety.^43^^,^^49^ Such a mechanism has been described for platelet-endothelial cell adhesion molecule (PECAM), which is not endocytosed, but still functionally sequestered away from the cell surface, but can be made available at the plasma membrane very rapidly.^52^ All these mechanisms together are likely to be able to produce the magnitude of membrane turnover required for producing the membrane-proximal EGF peak.

How can a ligand source be generated without continuous production of the ligand? Since an endocytic vesicle or a dent/pit in the membrane is filled by convection or flow,^53^ the process does not decrease the local ligand concentration. This endocytic or sequestration step is followed by very fast exocytosis of potentially the same vesicles to keep the membrane surface constant. One of the regulating mechanisms is membrane tension that is oppositely altered by endo- and exocytosis.^43^^,^^49^^,^^54^^,^^55^ As we pointed out previously, some kind of active biological work needs to be done to build the concentration gradient. We propose that shrinkage of endosomes or macropinosomes and the consequent re-exocytosis of concentrated endosomes can be the cause for the development of the membrane-proximal EGF concentration peak.^56^^,^^57^^,^^58^ Two-pore ion channels have been implicated in vesicle maturation and the shrinkage of endosomes.^40^^,^^41^ The fact that naringenin, a blocker of two-pore ion channels,^42^ significantly reduced the membrane-proximal EGF peak also supports the previous supposition. The sequestered (acid-strip resistant) pool of EGF just under the plasma membrane may correspond to the rapidly recycling/flickering endosomes generating the membrane-proximal EGF peak (Figure S8).

The locally elevated ligand concentration at the plasma membrane is more than an odd experimental observation since it has important consequences for cell biology. As shown in the current manuscript, it may significantly alter the concentration dependence of receptor activation making them look more affine and less cooperative (Figure 6). Therefore, the concentration dependence of ligand-induced responses, such as transmembrane signaling or receptor clustering, may differ between cellular systems that exhibit these effects and those that do not. It should be stressed that it is the apparent affinity and cooperativity of the receptor that may change due to the ligand concentration peaks if the ligand concentrations used for calculating these parameters are the bulk concentrations uncorrected for the reported effects. One way of rationalizing the increased apparent affinity is to consider that ligand concentrations close to the membrane are higher than the bulk concentration. If this effect is significant in the concentration range around the value of the dissociation constant, the fractional saturation of the receptor will be increased leading to the impression that the dissociation constant is smaller than in reality. As far as the decreased apparent cooperativity is concerned, one can rationalize this phenomenon by considering that the relative difference between the membrane proximal and bulk ligand concentrations decreases at high bulk ligand concentrations. Therefore, the ligand concentration range in which the receptor transitions between 10% and 90% saturation becomes wider (if saturation is plotted as a function of the bulk concentration). Since the width of this range is inversely related to cooperativity,^59^ this change will lead to an apparent decrease in cooperativity. Furthermore, responses of transmembrane receptors to ultralow ligand concentrations, attributed to preformed, higher-order signaling complexes, may also be partially caused by the membrane-proximal ligand concentration peak.^19^

Besides the membrane-proximal ligand concentration peak present in the immediate vicinity of the membrane, we also identified another one that is more distant from the membrane. This EGF concentration peak was clearly caused by a local increase in viscosity since it was inversely correlated with the local diffusion coefficient of EGF. This conclusion was corroborated by calculations showing that the observed local decrease in the diffusion coefficient of EGF is sufficient to account for the ligand concentration peak. The increased lifetime of a molecular rotor in this spatial range also supports that retarded diffusion in the membrane-distal ligand concentration peak is caused by higher local microviscosity (Figure 3C). While we could not unequivocally show the origin of the higher microviscosity, displacement of this concentration peak by a cocktail of collagenase and hyaluronidase in F1-4_ErbB2 cells suggests that it must be related to the extracellular matrix. In our CHO-derived cell lines, this EGF concentration peak was only present in ErbB2-expressing cells, but in other cell types, it may be ErbB2-independent. The correlation of this peak with ErbB2 is consistent with the known ability of ErbB2 to upregulate extracellular matrix production^60^^,^^61^ and by the contribution of ErbB2 to matrix stiffening by inducing collagen crosslinking.^62^

Hindered diffusion is the norm rather than the exception in biology. It has been shown that translational diffusion in the cytoplasm deviates from free diffusion with increasing molecular weight.^63^ However, molecules as small as inositol trisphosphate exhibit diffusion coefficient orders of magnitude smaller than expected based on their size, a phenomenon attributed to binding to immobile receptors.^64^ Lateral diffusion in the membrane is also a composite of unhindered, short-scale and hindered, long-scale diffusion components.^64^ A multitude of reasons can hinder lateral diffusion in biological systems. Binding to immobile structures,^65^^,^^66^ crossing of compartment barriers,^64^ a static network of the cytoskeleton or the extracellular matrix generating a sieving effect^63^ and the sheer density of obstacles^67^ have all been put forward to account for hindered diffusion. The latter two can be thought of as static and dynamic barriers, respectively, and they have been incorporated into the “fence and picket” model of lateral diffusion in the membrane.^68^ All these mechanisms might contribute to changes in local viscosity, a physical term obscuring the molecular background.

The membrane distal EGF concentration peak may have biological significance for several reasons. Even in our experiments it was superimposed on the membrane-proximal peak, and could, therefore, modify the concentration of EGF at the membrane. Depending on the density of the extracellular matrix, its influence on ligand concentrations at the plasma membrane may be even more profound. As a diffusion barrier and a potential reservoir of ligands, it may alter the temporal development of ligand concentrations *in vivo* and in experiments as it has been shown that growth factor binding to the extracellular matrix modifies the local concentration of ligands.^45^^,^^69^

Given the complexity of the methods for revealing the real, local ligand concentration at the membrane, it is unlikely that such measurements will become part of the everyday practice of ordinary cell biologists. When the aim of an experiment is to show a biological response to a ligand concentration established and known to work, the difference between the bulk and the membrane-proximal ligand concentration will only slightly alter the extent of the effect. However, interpretations of experiments aimed at revealing responses to ultralow ligand concentrations or at establishing dose-response relationships should take the presented membrane effects into consideration, and the actual, local ligand concentration should be measured and reported if models involving the bulk concentration fail to account for the experimental observations in a quantitative way.

In summary, we present evidence for the nonhomogeneous distribution of a peptide growth factor in the vicinity of the plasma membrane. We identified two different kinds of peaks regarding their origin, with one of them related to active membrane turnover and the other one to locally hindered diffusion. The concentration peaks significantly alter the ligand concentration at the plasma membrane compared to the bulk concentration altering dose dependent responses. We are certain that the wide-ranging implications of the presented results must not be overlooked when interpreting quantitative cell biological experiments involving stimulation of transmembrane receptors.

### Limitations of the study

The experiments and the theoretical models described in the current study reveal a strikingly nonhomogeneous distribution of ligand concentrations in the vicinity of the plasma membrane. Although the phenomena and the principles described potentially apply to any living biological systems from single-cell experiments to organisms, the fact whether such ligand concentrations gradients are present has to be established in every experimental system. Furthermore, the suggested molecular explanations might not apply to other cases. Given the complex nature of the experiments used for measuring the ligand concentrations, it is unlikely that such measurements will be widely applied in ordinary cell biological projects. However, the repercussions of these principles have to be kept in mind when interpreting any experimental result involving receptor-ligand interactions in a quantitative way.

## Resource availability

### Lead contact

Further information and requests for resources and reagents should be directed to and will be fulfilled by the lead contact, Peter Nagy (nagyp@med.unideb.hu).

### Materials availability

This study did not generate any new unique reagents.

### Data and code availability

• All data related to this article are available from the lead contact upon reasonable request.
• Code: Computer code used in this study is available at https://github.com/pet90d/Membran-effect-on-ligand-concentration.
• Any additional information needed to reanalyze the data reported in this paper is available from the lead author upon request.

## Acknowledgments

P.N. is supported by research grants from the 10.13039/501100018818National Research, Development and Innovation Office (Hungary; https://nkfih.gov.hu, K138075, ANN133421). J.S. is supported by the HUN-REN Hungarian Research Network (https://hun-ren.hu/). G.T. was supported by the EKÖP-24-4-I-DE-281 university research scholarship program of the Ministry for Culture and Innovation from the source of the 10.13039/501100012550National Research, Development and Innovation Fund. The funders had no role in study design, data collection and analysis, decision to publish, or preparation of the manuscript.

## Author contributions

Á.S. and G.T., investigation, writing—original draft, and visualization; T.S., G.M., and I.R., investigation; J.S., funding acquisition and writing—review and editing; P.N., conceptualization, writing—review and editing, supervision, and funding acquisition.

## Declaration of interests

The authors declare no competing interest.

## STAR★Methods

### Key resources table

**Table:** 

REAGENT or RESOURCE	SOURCE	IDENTIFIER
**Chemicals, peptides, and recombinant proteins**		

Tetramethylrhodamine-conjugated epidermal growth factor receptor (TAMRA-EGF)	ThermoFisher Scientific	Catalog number: E3481
Tetramethylrhodamine-conjugated bovine serum albumin (TAMRA-EGF)	ThermoFisher Scientific	Catalog number: A23016
8-Phenyl-BODIPY 505/515	MedChemExpress	Catalog number: HY-W089353
Tetramethylrhodamine	ThermoFisher Scientific	Catalog number: C300
Lipofectamine 2000	ThermoFisher Scientific	Catalog number: 11668500
Para-amino blebbistatin	Cayman Chemicals	Catalog number: 22699
Myristoylated dynamin inhibitory peptide (DIP)	Tocris-BioTechne	Catalog number: 1775
Latrunculin B	Sigma-Aldrich	Catalog number: L5288
Collagenase from *Clostridium histolyticum*	Sigma-Aldrich	Catalog number: C8176
Hyaluronidase from *Streptomyces hyalurolyticus*	Sigma-Aldrich	Catalog number: H1136
Naringenin	Sigma-Aldrich	Catalog number: N5893
Poly-L-lysine	Sigma-Aldrich	Catalog number: P1399

**Critical commercial assays**		

Qifikit	Agilent Technologies	Catalog number: K0078

**Experimental models: Cell lines**		

CHO	ATCC	Catalog number: CCL-61

**Recombinant DNA**		

pECFP-N1 plasmid	Clonetech	not available any more from Clonetech (Takara)
pEGFP-N3 plasmid	Clonetech	not available any more from Clonetech (Takara)

**Software and algorithms**		

MATLAB	Mathworks	n.a.
QuickFit 3	Jan Wolfgang Krieger, Jörg Langowski, German Cancer Research Center, Heidelberg, Germany	n.a.
SymPhoTime64	PicoQuant	n.a.

**Other**		

8-well chambered coverglass	Ibidi	Catalog number: 80826

### Experimental model and study participant details

#### Cell lines

F1-4 is a subline of CHO cells (American Type Culture Collection, Manassas, VA) stably transfected to express EGF receptor tagged with enhanced green fluorescent protein (EGFP) on the C-terminal.^30^ F1-4 cells were maintained at 37°C in the presence of 5% CO_2_ in Dulbecco’s Modified Eagle’s medium (DMEM) supplemented with 10% fetal calf serum and 50 μg/mL gentamicin. The number of passages of cells used for the experiments never exceeded 15. Cells were periodically tested for the presence of mycoplasma and authenticated by Eurofins Genomics (Ebersberg, Germany). The average expression level of EGFR on F1-4 cells, determined by flow cytometry using Qifikit, was ∼6⋅10^5^ receptors/cell, and these cells were found to express ErbB2 at undetectable levels using immunofluorescence labeling. F1-4_ErbB2 cells were generated from the F1-4 line by transiently transfecting it with ErbB2-ECFP (ErbB2 conjugated to enhanced cyan fluorescent protein in a pECFP-N1 plasmid, a kind gift from Donna J. Arndt-Jovin, Max Planck Institute for Multidisciplinary Sciences, Göttingen, Germany). For microscopic investigation of these cells, cells exhibiting both green and cyan fluorescence were selected ensuring that they expressed both EGFR and ErbB2. As usual for transient transfections, these cells exhibited a wide range of ErbB2 expressions. According to quantitation of protein expression by Qifikit, cells displaying bright cyan fluorescence, the ones we usually selected in a microscopic field of view, expressed 3–7⋅10^5^ ErbB2 receptors/cell.

### Method details

#### Treatment and fluorescence labeling of cells

For microscopic experiments cells were cultured on μ-slide 8-well chambered coverglass (Ibidi), and transfected, if required, by ErbB2-ECFP. Transfection was carried out using Lipofectamine 2000 according to the manufacturer’s specifications. Tetramethylrhodamine-conjugated EGF (TAMRA-EGF) was used for labeling cells and for measuring the concentration of the growth factor in the vicinity of the plasma membrane using FCS. In order to remove membrane-bound, non-internalized ligand from the membrane, cells were treated with acid strip buffer (200 mM acetic acid, 500 mM NaCl, pH 2.8) for 5 min on ice followed by immediate washing with Hank’s balanced salt solution twice. Latrunculin B, an inhibitor of actin polymerization, para-amino blebbistatin, a myosin inhibitor, and myristoylated dynamin inhibitory peptide (DIP) were used at a concentration of 5, 50 and 50 μM, respectively, to inhibit membrane turnover. Cells were incubated with these agents for 60 min at 37°C, and the agents were present at the same concentration during the whole duration of the experiments. In order to digest the extracellular matrix, cells were incubated with 20 μg/mL collagenase from *Clostridium histolyticum* and 20 U/mL hyaluronidase from *Streptomyces hyalurolyticus* for 90 min at 37°C. In order to inhibit the activity of two-pore ion channel 2 (TPC2),^42^ cells were pretreated with naringenin at a concentration of 1 mM for 30 min at 37°C, and the inhibitor was also present throughout the whole duration of the FCS measurement.

#### Measurement of viscosity with a BODIPY-based molecular rotor

Molecular rotors are fluorescent reporters in which the rotational freedom of a bond is limited in highly viscous environments resulting in an increased fluorescence lifetime.^33^^,^^34^ 8-Phenyl-BODIPY 505/515 was added to the medium of F1-4_ErbB2 cells and the fluorescence lifetime of the dye was measured above the cells using time-correlated single photon counting with a Nikon A1 Eclipse Ti2 confocal laser scanning microscope (Nikon, Tokyo) equipped with a Plan-Apochromat 60× water immersion objective (NA = 1.27) and a time-correlated single-photon counting upgrade kit (PicoQuant, Berlin, Germany). Pulsed excitation was achieved by a laser emitting ∼1.5-ps pulses at 485 nm at a repetition rate of 20 MHz. Fluorescence emission was measured with a PMA hybrid 40 photon-counting photomultiplier (PicoQuant) recording the emission between 505 and 535 nm. A double-exponential equation was fitted to the time-dependent fluorescence decay curves in SymPhoTime (PicoQuant) providing the lifetimes (*τ*_1_, *τ*_2_) and the amplitudes (*A*_1_, *A*_2_) of the two components from which an intensity-weighted lifetime (*τ*_eff_) was calculated according to the following equation:

(Equation 1) $$\tau_{eff}=\frac{\sum_{i}A_{i}\;{\tau_{i}}^{2}}{\sum_{i}A_{i}\;\tau_{i}}$$

#### Preparation of giant plasma membrane vesicles

Giant plasma membrane vesicles (GPMV) were produced by osmotic vesiculation.^35^^,^^70^ Briefly, cells were washed twice with hypotonic wash buffer (PBS diluted to 30% with distilled water) followed by a 12-h incubation with hypertonic vesiculation buffer (200 mM NaCl, 0.75 mM CaCl_2_, 5 mM KCl, 0.5 mM MgCl_2_, 100 mM bicine, pH 8.5) at 37°C. Release of the plasma membrane-derived vesicles was fostered by gentle tapping of the plate. The supernatant containing the GPMVs was collected, and 150 μL was added to a single well of an 8-well chambered coverglass pretreated with 0.1% (w/v) poly-L-lysine for 3 h to promote attachment of the vesicles to the glass surface.

#### Fluorescence correlation spectroscopy

FCS measurements were carried out at room temperature on a Nikon A1 Eclipse Ti2 confocal laser scanning microscope using a Plan Apo 60× water immersion objective (NA = 1.27). TAMRA was excited by the 561-nm laser, and its emission was detected through a 594-nm longpass filter by a single photon counting detector (PicoQuant). Fluorescence intensity was recorded in 8-s runs 10 times at distances of 1–100 μm above the plasma membrane of attached cells, and autocorrelation curves were calculated with the SymPhoTime64 software tool (PicoQuant) at 200 time points from 300 ns to 1 s with a quasi-logarithmic timescale.

### Quantification and statistical analysis

#### Evaluation of fluorescence correlation spectroscopy experiments

After averaging 10 autocorrelation curves, the following equation containing a triplet term and a single diffusion component was fitted to the experimental data:

(Equation 2) $$G\left(t\right)=1+\frac{1}{N}\frac{1-T\left(1-e^{-\frac{t}{\tau_{triplet}}}\right)}{1-T}\frac{1}{\left(1+\frac{t}{\tau_{D}}\right)\sqrt{1+\frac{t}{\kappa^{2}\;\tau_{D}}}}$$

where *N* is the average number of fluorescent particles in the confocal detection volume, *T* is the fraction of fluorophores in the triplet state, *κ* is the beam shape parameter, *τ*_triplet_ is the triplet correlation time and *τ*_D_ is the diffusion correlation time. The number of particles per confocal volume was converted to molar concentrations using the following expression for the confocal volume (*V*_C_):

(Equation 3) $$V_{C}=\pi^{3/2}\kappa \;\omega^{3}$$

The diffusion coefficient (*D*) was determined from the diffusion correlation time according to the following equation:

(Equation 4) $$D=\frac{\omega^{2}}{4\tau_{D}}$$

where *ω* is the distance between the beam center and the point where the optical intensity of the laser drops to e^−2^ of the maximum value. The beam radius was determined by measuring the autocorrelation curve of free Alexa Fluor 546, entering the known diffusion coefficient of Alexa Fluor 546 (341 μm^2^/s at 25°C)^71^ into Equation 2 and leaving *ω* as a fitted parameter. Autocorrelation curves were fitted with QuickFit 3. The FCS results shown in the manuscript are based on the evaluation of at least ten curves.

#### Calculation of the concentration gradient modified by flow

Let us assume a spherically symmetric geometry with a cell of radius *a* placed at the center of symmetry. While this assumption obviously does not perfectly describe the system of an adherent monolayer of cultured cells, more detailed and realistic models would have required a lot of parameters, which would have made the model less tractable mathematically and practically. The presence of a steady-state concentration gradient implies that the time-dependent change in the concentration gradient is zero. Therefore, Fick’s second law takes the following form:

(Equation 5) $$D\;\nabla^{2}c=0$$

Let us rewrite the equation above in spherical coordinates and let us include only the radial coordinate due to the assumed spherical symmetry:

(Equation 6) $$\frac{D}{r^{2}}\frac{\partial \left(r^{2}\frac{\partial c}{\partial r}\right)}{\partial r}=0$$

It is worth pointing out that the solution of this differential equation along the radial direction will result in a perfect description of the concentration profile in three dimensions since the concentration is independent of the polar and azimuthal angles (due to the spherical symmetry). The requirement for the derivative to be zero implies that the term in brackets must be constant:

(Equation 7) $$r^{2}\frac{\partial c}{\partial r}=K_{1}$$

The general solution of this differential equation takes the following form:

(Equation 8) $$c=-\frac{K_{1}}{r}+K_{2}$$

Let us formulate two boundary conditions: (i) the concentration of the ligand at r→∞ is equal to the bulk concentration, *c*(∞) = *c*_0_; (ii) the concentration of the ligand at the membrane is *c*(*a*) = *c*_m_. By applying these two boundary conditions, the concentration profile of the ligand can be calculated:

(Equation 9) $$\begin{array}{l} K_{1}=a\left(c_{0}-c_{m}\right)\\ K_{2}=c_{0} \end{array}\}c=\frac{a\left(c_{m}-c_{0}\right)}{r}+c_{0}$$

The concentration profile generated by the previous equation is shown in Figure 2B.

The presence of this concentration gradient in the immediate vicinity of the plasma membrane not coinciding with a viscosity gradient implies that there is a constant flow of ligand from areas of high concentration to areas of low concentration. The flux at the cell membrane can be calculated according to Fick’s first equation:

(Equation 10) $$J\left(a\right)=-D{\frac{\partial c}{\partial r}|}_{a}=D\frac{c_{m}-c_{0}}{a}$$

The unit of *c*_m_ and *c*_0_ are number of particles/confocal volume. Assuming the confocal volume is 0.16 μm^3^, let us convert their unit to number of particles/μm^3^: c_m_ = 18.75 1/μm^3^, *c*_0_ = 6.25 1/μm^3^. Using these values and a diffusion coefficient of *D* = 150 μm^2^/s, the ligand flux from within the cell to the extracellular space is 625 1/(μm^2^ s), i.e., 625 EGF molecules must be exocytosed in a second in a membrane area of 1 μm^2^ to sustain the concentration gradient.

#### Calculation of the local concentration gradient due to locally hindered diffusion

Observation of a local minimum in the diffusion coefficient of TAMRA-EGF spatially coinciding with a local maximum in TAMRA-EGF concentration implied that a stable viscosity gradient is present. Such a spatially stable, local minimum of diffusivity is known to generate a concentration gradient, and we wanted to find out if the magnitude of this expected concentration gradient is equivalent to the observed one. In such an inhomogeneous space, diffusion is described by the Fokker-Planck equation, which assumes the following form for a steady-state condition:

(Equation 11) $$\nabla^{2}\left(D\;c\right)=0$$

where *c* and *D* are the concentration and the diffusion coefficient of TAMRA-EGF, respectively. Rewriting the Laplacian operator in spherical coordinates and considering only the radial component result in the following equation:

(Equation 12) $$\frac{1}{r^{2}}\frac{\partial \left(r^{2}\frac{\partial \left(D\;c\right)}{\partial r}\right)}{\partial r}=0$$

For the derivate to be zero, the term in parentheses has to be constant:

(Equation 13) $$r^{2}\frac{\partial \left(D\;c\right)}{\partial r}=K_{3}$$

The measured dependence of *D* on *r* was approximated by a power function (Figure S3):

(Equation 14) $$D\left(r\right)=p_{1}{\left(r-p_{2}\right)}^{2}+p_{3}$$

with *p*_1_ = 0.32, *p*_2_ = 23.47, *p*_3_ = 91.32. Solution of differential Equation 13 after substituting the aforementioned distance dependence of the diffusion coefficient results in the following general solution:

(Equation 15) $$c=\frac{-K_{3}+r\;K_{4}}{\left(p_{3}+p_{1}{\left(p_{2}-r\right)}^{2}\right)r}$$

Applying the Dirichlet boundary conditions of *c*(35) = *c*(13) = 2, i.e., the concentrations of TAMRA-EGF measured by FCS at the indicated positions, the constants assume the values of *K*_3_ = 291.2, *K*_4_ = 275.56. *c*(35) and *c*(13) are the concentrations at *r* = 35 μm and *r* = 13 μm from the center of symmetry, which correspond to a distance of 25 μm and 3 μm from the membrane, respectively, due to the assumption that the radius of the cell is 10 μm. Equation 15 with the above constants produces a concentration gradient shown in Figure 2A whose magnitude is similar to the experimentally observed one (Figure 1B). Therefore, we concluded that the viscosity gradient (local minimum in the diffusion coefficient) sufficiently explains the experimentally observed membrane-distal concentration peak of TAMRA-EGF in F1-4_ErbB2 cells.

#### Simulation of the concentration gradient generated by binding and exocytosis

It has been suggested that a binding equilibrium at the membrane, i.e., equilibrium between free ligands and ligands bound specifically to receptors or non-specifically to the membrane, can explain the higher local ligand concentration at the membrane.^22^ In order to check the effect of the aforementioned binding equilibrium on the local ligand concentration at the membrane, a system of partial differential equations was established describing the diffusion of the ligand and its binding to cell surface receptors. Due to the assumed spherical symmetry, a 1-D model involving the radial component of Laplace operator in spherical coordinates was sufficient to describe the system:

(Equation 16) $$\begin{array}{c} \frac{\partial L}{\partial t}=\frac{D}{r^{2}}\frac{\partial \left(r^{2}\frac{\partial L}{\partial r}\right)}{\partial r}+k_{off}\;LR-k_{on}\;L\;R\\ \frac{\partial LR}{\partial t}=-k_{off}\;LR+k_{on}\;L\;R\\ \frac{\partial R}{\partial t}=k_{off}\;LR-k_{on}\;L\;R \end{array}$$

in which *L*, *LR* and *R* correspond to the concentration of the ligand, the liganded receptor and the unliganded receptor, respectively, *D* is the diffusion coefficient, *r* is the distance from the origin, *k*_on_ and *k*_off_ are the association and dissociation rate constants, respectively.

The equation system was simulated with the “pdepe” function of MATLAB solving 1-D partial differential equations of the following form:

(Equation 17) $$c\frac{\partial u}{\partial t}=x^{-m}\frac{\partial \left(x^{m}f\right)}{\partial r}+s$$

where *c*, *f* and *s* are functions of position (*x*), time (*t*), the solution (*u*) and its spatial derivative (d*u*/d*x*). *m* defines the symmetry of the system, which was chosen to be 2 corresponding to spherical symmetry. Since there are three equations in the equation set, the parameters are 3 × 1 column vectors:

(Equation 18) $$c=\left[\begin{array}{l} 1\\ 1\\ 1 \end{array}\right],f=\left[\begin{array}{l} D\partial u_{1}/\partial r\\ 0\\ 0 \end{array}\right],s=\left[\begin{array}{l} k_{off}\;LR-k_{on}\;L\;R\\ -k_{off}\;LR+k_{on}\;L\;R\\ k_{off}\;LR-k_{on}\;L\;R \end{array}\right]$$

where *u*_1_ denotes the ligand concentration, *u*_2_ and *u*_3_ correspond to LR and R, respectively. The simulation was performed outside a sphere (cell) whose radius was 10 μm, and the simulated space ended at 100 μm. The results of several simulations performed using the geometry described above are shown in Figure S5.

(1) In the first simulation, the ligand concentration was 10 nM in the whole extracellular space, and unliganded EGF receptor was added to the plasma membrane at the beginning of the simulation. The receptor concentration was chosen so that ligand depletion was approximately 10% after reaching equilibrium. The model simulates what kind of effect the introduction of receptors to the system has on the homogeneous distribution of the ligand. The diffusion coefficient was set to 150 μm^2^/s in accordance with our measurements, whereas the association and dissociation rate constants were 0.0023 1/(nM s) and 0.003 1/s, respectively.^72^^,^^73^ The partial differential equation set was solved numerically with a zero-flux boundary condition resulting in the solution shown in Figure S5A. Introduction of unliganded EGF receptor to the plasma membrane results in a transient, negative concentration peak. However, no ligand concentration gradient is present once the dissociation equilibrium is reached.
(2) The system simulated in Figure S5B had unliganded EGF receptors at the plasma membrane and free EGF at a concentration of 40 nM between 80 and 90 μm away from the membrane. The ligand diffused toward the membrane and bound to unliganded EGF receptors during the simulation. A transient negative concentration peak was present at the membrane, but after the establishment of equilibrium, the concentration of free EGF was homogeneous in the whole system.
(3) At the beginning of the simulation, a homogeneous free EGF concentration was present in the extracellular space, and a binding equilibrium was established between free ligand and EGF receptors at the membrane (Figure S5C). Liganded EGF receptor was added to the plasma membrane at *t* = 0 (“exocytosis”), which resulted in the appearance of a transient, positive EGF concentration peak of the membrane.
(4) The system simulated in Figure S5D was almost identical to the one shown in Figure S5C, but instead of a single exocytosis of liganded EGF receptor, EGF was continuously added to the system at the plasma membrane. The concentration of free ligand at the far end of the geometry was kept constant by applying a Dirichlet boundary condition. This continuous flux of EGF from the membrane resulted in a permanent, positive concentration peak in the vicinity of the plasma membrane.

The MATLAB Live Script performing the simulations are available at the following GitHub repository: https://github.com/pet90d/Membran-effect-on-ligand-concentration.

## References

[1] Yin H., Flynn A.D. (2016). Drugging membrane protein interactions. Annu. Rev. Biomed. Eng..

[2] Bethani I., Skånland S.S., Dikic I., Acker-Palmer A. (2010). Spatial organization of transmembrane receptor signalling. EMBO J..

[3] Low-Nam S.T., Lidke K.A., Cutler P.J., Roovers R.C., van Bergen en Henegouwen P.M.P., Wilson B.S., Lidke D.S. (2011). ErbB1 dimerization is promoted by domain co-confinement and stabilized by ligand binding. Nat. Struct. Mol. Biol..

[4] Kovács T., Zákány F., Nagy P. (2022). It takes more than two to tango: complex, hierarchal, and membrane-modulated interactions in the regulation of receptor tyrosine kinases. Cancers (Basel).

[5] Moriki T., Maruyama H., Maruyama I.N. (2001). Activation of preformed EGF receptor dimers by ligand-induced rotation of the transmembrane domain. J. Mol. Biol..

[6] Gadella T.W., Jovin T.M. (1995). Oligomerization of epidermal growth factor receptors on A431 cells studied by time-resolved fluorescence imaging microscopy. A stereochemical model for tyrosine kinase receptor activation. J. Cell Biol..

[7] Szabó A., Horváth G., Szöllosi J., Nagy P. (2008). Quantitative characterization of the large-scale association of ErbB1 and ErbB2 by flow cytometric homo-FRET measurements. Biophys. J..

[8] Clayton A.H.A., Tavarnesi M.L., Johns T.G. (2007). Unligated epidermal growth factor receptor forms higher order oligomers within microclusters on A431 cells that are sensitive to tyrosine kinase inhibitor binding. Biochemistry.

[9] Needham S.R., Roberts S.K., Arkhipov A., Mysore V.P., Tynan C.J., Zanetti-Domingues L.C., Kim E.T., Losasso V., Korovesis D., Hirsch M. (2016). EGFR oligomerization organizes kinase-active dimers into competent signalling platforms. Nat. Commun..

[10] Zanetti-Domingues L.C., Korovesis D., Needham S.R., Tynan C.J., Sagawa S., Roberts S.K., Kuzmanic A., Ortiz-Zapater E., Jain P., Roovers R.C. (2018). The architecture of EGFR's basal complexes reveals autoinhibition mechanisms in dimers and oligomers. Nat. Commun..

[11] Tao R.H., Maruyama I.N. (2008). All EGF(ErbB) receptors have preformed homo- and heterodimeric structures in living cells. J. Cell Sci..

[12] Karunagaran D., Tzahar E., Beerli R.R., Chen X., Graus-Porta D., Ratzkin B.J., Seger R., Hynes N.E., Yarden Y. (1996). ErbB-2 is a common auxiliary subunit of NDF and EGF receptors: implications for breast cancer. EMBO J..

[13] Freed D.M., Bessman N.J., Kiyatkin A., Salazar-Cavazos E., Byrne P.O., Moore J.O., Valley C.C., Ferguson K.M., Leahy D.J., Lidke D.S., Lemmon M.A. (2017). EGFR ligands differentially stabilize receptor dimers to specify signaling kinetics. Cell.

[14] Hajdu T., Váradi T., Rebenku I., Kovács T., Szöllösi J., Nagy P. (2020). Comprehensive model for epidermal growth factor receptor ligand binding involving conformational states of the extracellular and the kinase domains. Front. Cell Dev. Biol..

[15] Schmick M., Bastiaens P.I.H. (2014). The interdependence of membrane shape and cellular signal processing. Cell.

[16] Care B.R., Soula H.A. (2013). Receptor clustering affects signal transduction at the membrane level in the reaction-limited regime. Phys. Rev. E Stat. Nonlin. Soft. Matter. Phys..

[17] Goldstein B., Posner R.G., Torney D.C., Erickson J., Holowka D., Baird B. (1989). Competition between solution and cell surface receptors for ligand. Dissociation of hapten bound to surface antibody in the presence of solution antibody. Biophys. J..

[18] Vauquelin G., Charlton S.J. (2010). Long-lasting target binding and rebinding as mechanisms to prolong in vivo drug action. Br. J. Pharmacol..

[19] Civciristov S., Ellisdon A.M., Suderman R., Pon C.K., Evans B.A., Kleifeld O., Charlton S.J., Hlavacek W.S., Canals M., Halls M.L. (2018). Preassembled GPCR signaling complexes mediate distinct cellular responses to ultralow ligand concentrations. Sci. Signal..

[20] Gillespie D.T. (2007). Stochastic simulation of chemical kinetics. Annu. Rev. Phys. Chem..

[21] Rosenfeld S. (2011). Mathematical descriptions of biochemical networks: stability, stochasticity, evolution. Prog. Biophys. Mol. Biol..

[22] Gherbi K., Briddon S.J., Charlton S.J. (2018). Micro-pharmacokinetics: quantifying local drug concentration at live cell membranes. Sci. Rep..

[23] Sykes D.A., Parry C., Reilly J., Wright P., Fairhurst R.A., Charlton S.J. (2014). Observed drug-receptor association rates are governed by membrane affinity: the importance of establishing "micro-pharmacokinetic/pharmacodynamic relationships" at the beta2-adrenoceptor. Mol. Pharmacol..

[24] Anderson G.P., Lindén A., Rabe K.F. (1994). Why are long-acting beta-adrenoceptor agonists long-acting?. Eur. Respir. J..

[25] Wu Y., Han B., Li Y., Munro E., Odde D.J., Griffin E.E. (2018). Rapid diffusion-state switching underlies stable cytoplasmic gradients in the Caenorhabditis elegans zygote. Proc. Natl. Acad. Sci. USA.

[26] Bressloff P.C., Lawley S.D., Murphy P. (2019). Protein concentration gradients and switching diffusions. Phys. Rev. E.

[27] Stapornwongkul K.S., Vincent J.P. (2021). Generation of extracellular morphogen gradients: the case for diffusion. Nat. Rev. Genet..

[28] Rangamani P., Lipshtat A., Azeloglu E.U., Calizo R.C., Hu M., Ghassemi S., Hone J., Scarlata S., Neves S.R., Iyengar R. (2013). Decoding information in cell shape. Cell.

[29] Tzahar E., Waterman H., Chen X., Levkowitz G., Karunagaran D., Lavi S., Ratzkin B.J., Yarden Y. (1996). A hierarchical network of interreceptor interactions determines signal transduction by Neu differentiation factor/neuregulin and epidermal growth factor. Mol. Cell Biol..

[30] Brock R., Hamelers I.H., Jovin T.M. (1999). Comparison of fixation protocols for adherent cultured cells applied to a GFP fusion protein of the epidermal growth factor receptor. Cytometry.

[31] Stefferson M.W., Norris S.L., Vernerey F.J., Betterton M.D., Hough L.E. (2017). Effects of soft interactions and bound mobility on diffusion in crowded environments: a model of sticky and slippery obstacles. Phys. Biol..

[32] Sullivan K.D., Brown E.B. (2011). Multiphoton fluorescence recovery after photobleaching in bounded systems. Phys. Rev..

[33] Michels L., Gorelova V., Harnvanichvech Y., Borst J.W., Albada B., Weijers D., Sprakel J. (2020). Complete microviscosity maps of living plant cells and tissues with a toolbox of targeting mechanoprobes. Proc. Natl. Acad. Sci. USA.

[34] Vysniauskas A., Lopez-Duarte I., Duchemin N., Vu T.T., Wu Y., Budynina E.M., Volkova Y.A., Pena Cabrera E., Ramirez-Ornelas D.E., Kuimova M.K. (2017). Exploring viscosity, polarity and temperature sensitivity of BODIPY-based molecular rotors. Phys. Chem. Chem. Phys..

[35] Sarabipour S., Chan R.B., Zhou B., Di Paolo G., Hristova K. (2015). Analytical characterization of plasma membrane-derived vesicles produced via osmotic and chemical vesiculation. Biochim. Biophys. Acta.

[36] Peng G.E., Wilson S.R., Weiner O.D. (2011). A pharmacological cocktail for arresting actin dynamics in living cells. Mol. Biol. Cell.

[37] Stein B.S., Bensch K.G., Sussman H.H. (1984). Complete inhibition of transferrin recycling by monensin in K562 cells. J. Biol. Chem..

[38] Basu S.K., Goldstein J.L., Anderson R.G., Brown M.S. (1981). Monensin interrupts the recycling of low density lipoprotein receptors in human fibroblasts. Cell.

[39] Mollenhauer H.H., Morré D.J., Rowe L.D. (1990). Alteration of intracellular traffic by monensin; mechanism, specificity and relationship to toxicity. Biochim. Biophys. Acta.

[40] Freeman S.A., Uderhardt S., Saric A., Collins R.F., Buckley C.M., Mylvaganam S., Boroumand P., Plumb J., Germain R.N., Ren D., Grinstein S. (2020). Lipid-gated monovalent ion fluxes regulate endocytic traffic and support immune surveillance. Science.

[41] Wang X., Zhang X., Dong X.P., Samie M., Li X., Cheng X., Goschka A., Shen D., Zhou Y., Harlow J. (2012). TPC proteins are phosphoinositide- activated sodium-selective ion channels in endosomes and lysosomes. Cell.

[42] Pafumi I., Festa M., Papacci F., Lagostena L., Giunta C., Gutla V., Cornara L., Favia A., Palombi F., Gambale F. (2017). Naringenin impairs two-pore channel 2 activity and inhibits VEGF-induced angiogenesis. Sci. Rep..

[43] Smith S.M., Renden R., von Gersdorff H. (2008). Synaptic vesicle endocytosis: fast and slow modes of membrane retrieval. Trends Neurosci..

[44] Grecco H.E., Schmick M., Bastiaens P.I.H. (2011). Signaling from the living plasma membrane. Cell.

[45] Lander A.D., Nie Q., Wan F.Y.M. (2002). Do morphogen gradients arise by diffusion?. Dev. Cell.

[46] Pero J.K., Haas E.M., Thompson N.L. (2006). Size dependence of protein diffusion very close to membrane surfaces: measurement by total internal reflection with fluorescence correlation spectroscopy. J. Phys. Chem. B.

[47] Fradin C., Abu-Arish A., Granek R., Elbaum M. (2003). Fluorescence correlation spectroscopy close to a fluctuating membrane. Biophys. J..

[48] Gasman S., Chasserot-Golaz S., Malacombe M., Way M., Bader M.F. (2004). Regulated exocytosis in neuroendocrine cells: a role for subplasmalemmal Cdc42/N-WASP-induced actin filaments. Mol. Biol. Cell.

[49] Ren L., Mellander L.J., Keighron J., Cans A.S., Kurczy M.E., Svir I., Oleinick A., Amatore C., Ewing A.G. (2016). The evidence for open and closed exocytosis as the primary release mechanism. Q. Rev. Biophys..

[50] Sun J.Y., Wu L.G. (2001). Fast kinetics of exocytosis revealed by simultaneous measurements of presynaptic capacitance and postsynaptic currents at a central synapse. Neuron.

[51] Watanabe S., Boucrot E. (2017). Fast and ultrafast endocytosis. Curr. Opin. Cell Biol..

[52] Maxfield F.R., McGraw T.E. (2004). Endocytic recycling. Nat. Rev. Mol. Cell Biol..

[53] Lowengrub J., Allard J., Aland S. (2016). Numerical simulation of endocytosis: Viscous flow driven by membranes with non-uniformly distributed curvature-inducing molecules. J. Comput. Phys..

[54] Sheetz M.P. (2001). Cell control by membrane-cytoskeleton adhesion. Nat. Rev. Mol. Cell Biol..

[55] Ogunmowo T.H., Jing H., Raychaudhuri S., Kusick G.F., Imoto Y., Li S., Itoh K., Ma Y., Jafri H., Dalva M.B. (2023). Membrane compression by synaptic vesicle exocytosis triggers ultrafast endocytosis. Nat. Commun..

[56] Buckley C.M., King J.S. (2017). Drinking problems: mechanisms of macropinosome formation and maturation. FEBS J..

[57] Podinovskaia M., Prescianotto-Baschong C., Buser D.P., Spang A. (2021). A novel live-cell imaging assay reveals regulation of endosome maturation. eLife.

[58] Chadwick S.R., Wu J.Z., Freeman S.A. (2021). Solute transport controls membrane tension and organellar volume. Cell. Physiol. Biochem..

[59] Cattoni D.I., Chara O., Kaufman S.B., González Flecha F.L. (2015). Cooperativity in binding processes: new insights from phenomenological modeling. PLoS One.

[60] Ueda Y., Wang S., Dumont N., Yi J.Y., Koh Y., Arteaga C.L. (2004). Overexpression of HER2 (erbB2) in human breast epithelial cells unmasks transforming growth factor beta-induced cell motility. J. Biol. Chem..

[61] Jeon M., Lee J., Nam S.J., Shin I., Lee J.E., Kim S. (2015). Induction of fibronectin by HER2 overexpression triggers adhesion and invasion of breast cancer cells. Exp. Cell Res..

[62] Levental K.R., Yu H., Kass L., Lakins J.N., Egeblad M., Erler J.T., Fong S.F.T., Csiszar K., Giaccia A., Weninger W. (2009). Matrix crosslinking forces tumor progression by enhancing integrin signaling. Cell.

[63] Arrio-Dupont M., Foucault G., Vacher M., Devaux P.F., Cribier S. (2000). Translational diffusion of globular proteins in the cytoplasm of cultured muscle cells. Biophys. J..

[64] Lagerholm B.C., Andrade D.M., Clausen M.P., Eggeling C. (2017). Convergence of lateral dynamic measurements in the plasma membrane of live cells from single particle tracking and STED-FCS. J. Phys. D Appl. Phys..

[65] Dickinson G.D., Ellefsen K.L., Dawson S.P., Pearson J.E., Parker I. (2016). Hindered cytoplasmic diffusion of inositol trisphosphate restricts its cellular range of action. Sci. Signal..

[66] Brazda P., Szekeres T., Bravics B., Tóth K., Vámosi G., Nagy L. (2011). Live-cell fluorescence correlation spectroscopy dissects the role of coregulator exchange and chromatin binding in retinoic acid receptor mobility. J. Cell Sci..

[67] Brown F.L., Leitner D.M., McCammon J.A., Wilson K.R. (2000). Lateral diffusion of membrane proteins in the presence of static and dynamic corrals: suggestions for appropriate observables. Biophys. J..

[68] Ritchie K., Iino R., Fujiwara T., Murase K., Kusumi A. (2003). The fence and picket structure of the plasma membrane of live cells as revealed by single molecule techniques (Review). Mol. Membr. Biol..

[69] Sawicka K.M., Seeliger M., Musaev T., Macri L.K., Clark R.A.F. (2015). Fibronectin interaction and enhancement of growth factors: importance for wound healing. Adv. Wound Care.

[70] Del Piccolo N., Placone J., He L., Agudelo S.C., Hristova K. (2012). Production of plasma membrane vesicles with chloride salts and their utility as a cell membrane mimetic for biophysical characterization of membrane protein interactions. Anal. Chem..

[71] Petrasek Z., Schwille P. (2008). Precise measurement of diffusion coefficients using scanning fluorescence correlation spectroscopy. Biophys. J..

[72] French A.R., Tadaki D.K., Niyogi S.K., Lauffenburger D.A. (1995). Intracellular trafficking of epidermal growth factor family ligands is directly influenced by the pH sensitivity of the receptor/ligand interaction. J. Biol. Chem..

[73] DeWitt A., Iida T., Lam H.Y., Hill V., Wiley H.S., Lauffenburger D.A. (2002). Affinity regulates spatial range of EGF receptor autocrine ligand binding. Dev. Biol..

