# Supplementary material for: Local ligand concentration gradients induced by the plasma membrane
Source: iScience. 2025 Jun 19;28(7):112954. doi: 10.1016/j.isci.2025.112954 (PMC12273584; doi:10.1016/j.isci.2025.112954)
Supplement: Document S1. Figures S1–S11 [file mmc1.pdf]

**iScience, Volume *28***

## Supplemental information

# Local ligand concentration gradients induced by the plasma membrane

**Ágnes Szabó, Gabriella Tóth, Tímea Szatmári, Gábor Mocsár, István Rebenku, János Szöllősi, and Peter Nagy**

## Supplementary Figures

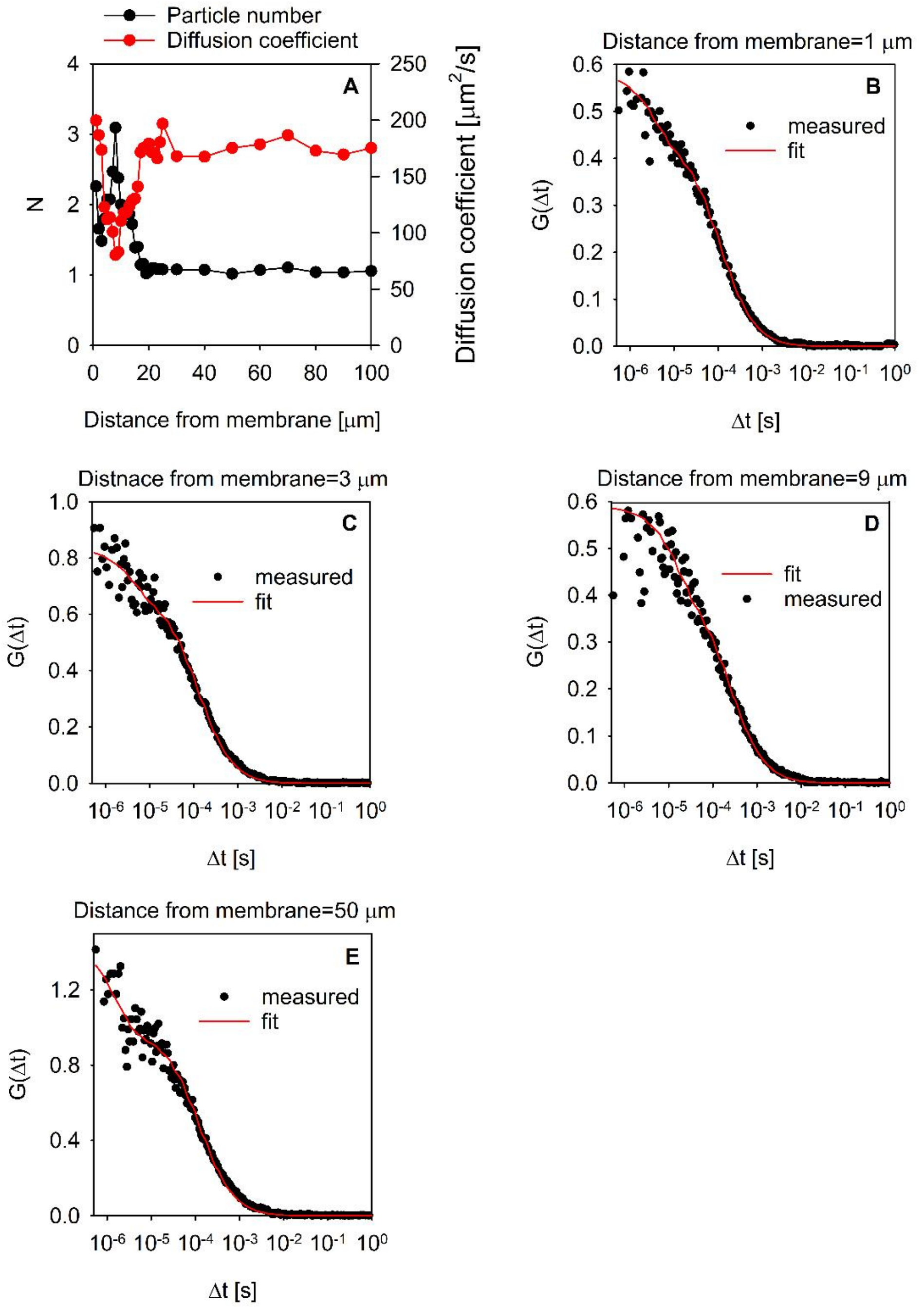


**Supplementary Figure 1. Representative FCS curves and their fits.** F1-4_ErbB2 cells were incubated in the presence of 10 nM TAMRA-EGF, and FCS was performed to determine the particle number and the diffusion coefficient of TAMRA-EGF, which are plotted for a single experiment in panel A. Original FCS curves recorded at the indicated distances from the membrane and their fits by equation (2) are shown in B-E.

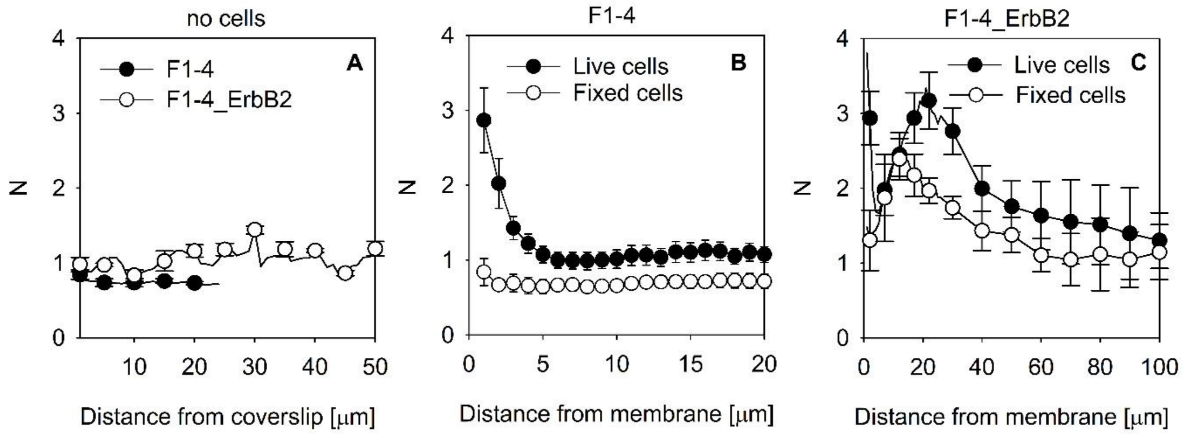


**Supplementary Figure 2. Ligand concentration gradients in the absence of cells, and in the presence of live and fixed cells.** F1-4 and F1-4_ErbB2 cells were incubated with 10 nM TAMRA-EGF, and the ligand concentrations were measured in a cell-free area as a function of distance from the coverslip (A), and above cells (black curves in B and C). Alternatively, cells were fixed, washed free of the fixative followed by incubation in the presence of 10 nM TAMRA-EGF. The concentration of the ligand as a function of distance from the membrane is shown by the empty symbols in B and C.

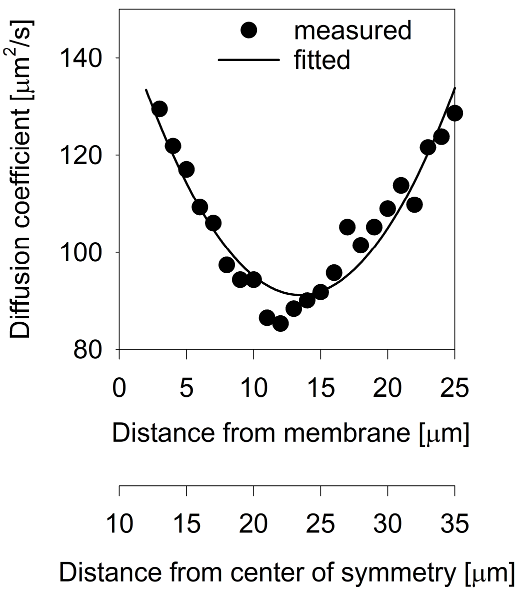


**Supplementary Figure 3. Fitting of the dependence of the diffusion coefficient on the distance from the membrane.** The positive peak of TAMRA-EGF concentration coincided with a negative peak of the diffusion coefficient of TAMRA-EGF for F1-4_ErbB2 cells (Fig. 1B). Analytical description of the distance dependence of the diffusion coefficient was required for calculation of the concentration gradient generated by the local minimum of the diffusion coefficient. The following quadratic function was found to fit the experimentally determined diffusion coefficients adequately:

$$D(r) = 0.32(r - 23.47)^2 + 91.32$$

where *r* is the distance from the center of symmetry and *D*(r) is the distance-dependent diffusion coefficient. Since the cell was assumed to be a sphere with a radius of 10 μm, the distance from the center of spherical symmetry is by 10 μm more than the distance from the membrane, the usual quantity displayed on the X-axis throughout the manuscript.

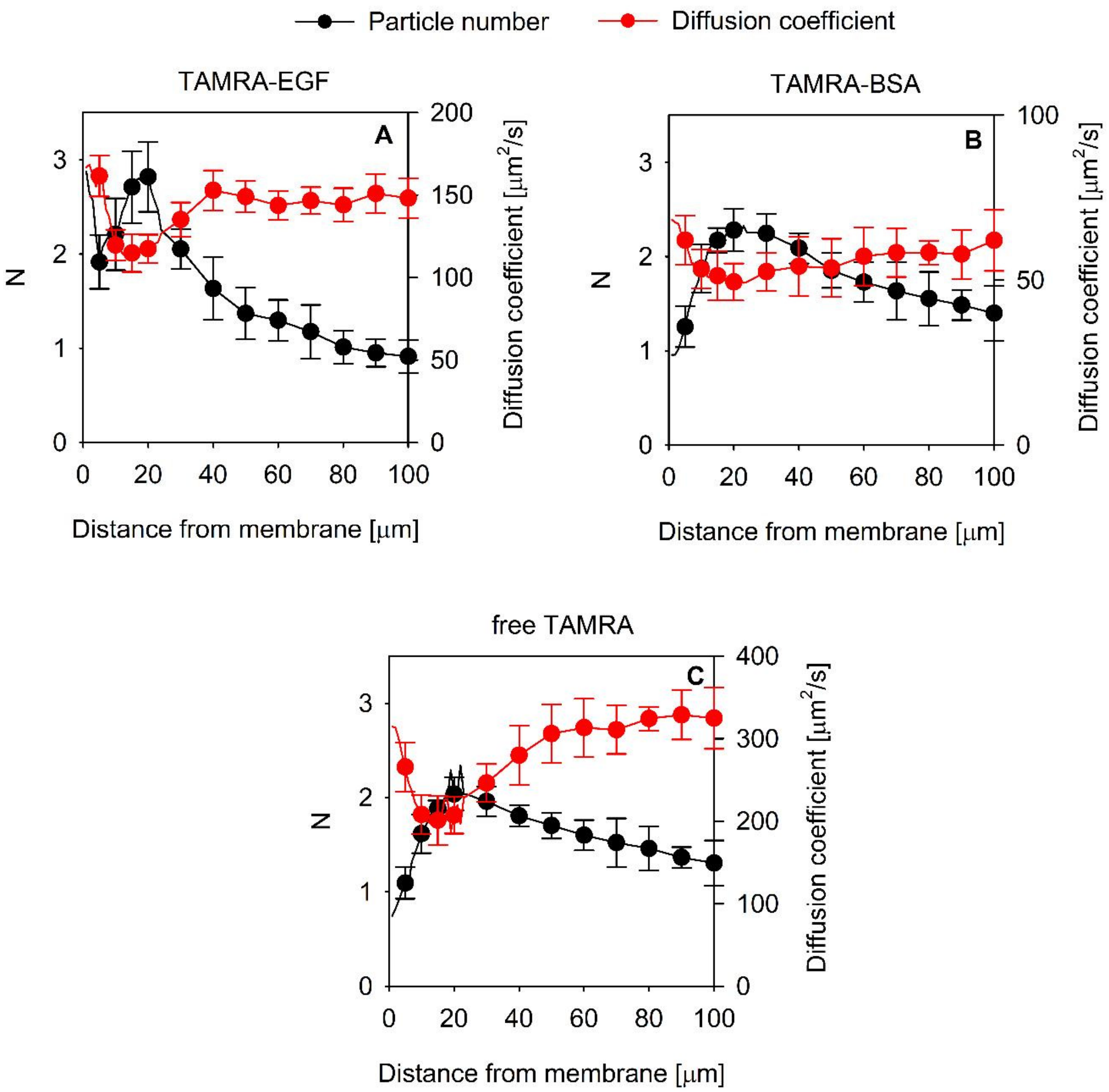


**Supplementary Figure 4. Comparison of the correlation between the membrane-distant concentration peak and the local minimum of the diffusion coefficient for several different molecules.** F1-4_ErbB2 cells were incubated in the presence of 10 nM TAMRA-EGF (A), TAMRA-BSA (B) or TAMRA (C) and FCS measurements were performed to determine the number of particles per confocal volume (*N*) and the diffusion coefficient. The error bars represent the standard deviation.

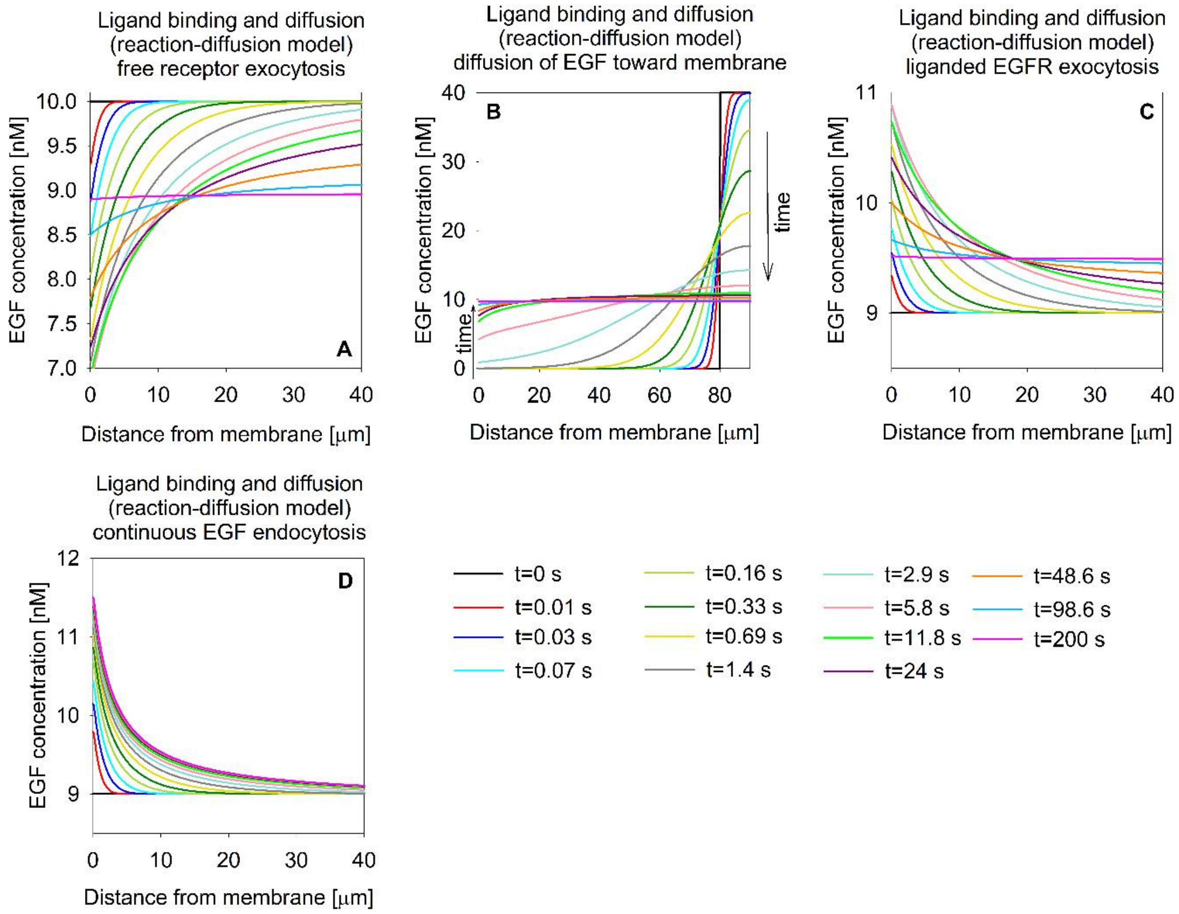


**Supplementary Figure 5. Simulation of the concentration profile of free ligand in the vicinity of receptors expressed in the plasma membrane.**

(A) A sphere with a radius of 10 µm was placed in the center of a spherically symmetric geometry with an outer radius of 100 µm. A homogeneous, 10 nM concentration of EGF was added to the simulated geometry, and at the beginning of the simulation, ligand-free, immobile receptors were placed on the surface of the 10 µm-radius sphere ("cell membrane"). The number of receptors was determined retrospectively so that the extent of ligand depletion was ~10% (final, equilibrium concentration of free EGF in the bulk solution is ~9 nM). The system between *r*=10 µm and *r*=100 µm was simulated with no flux boundary conditions on a mesh with 0.1 µm divisions using the 'pdepe' function of MATLAB. The association and dissociation rate constants were assumed to be 0.0023 $nM^{-1}$ $s^{-1}$ and 0.003 $s^{-1}$, respectively, resulting in a dissociation constant of ~1.3 nM[1,2]. The diffusion coefficient of EGF was set to 150 $\mu m^2/s$ in accordance with our FCS measurements. The graph shows the EGF concentration profile at logarithmically-spaced time points up to 200 seconds when equilibrium was reached. While a transient, negative concentration peak is present adjacent

to the membrane, the concentration of EGF in the system is homogeneous after reaching equilibrium.

(B) A system similar to the one described in A was simulated, but this time EGF was added between 80 and 90 μm from the cell at the beginning of the simulation, and the ligand was allowed to diffuse toward the plasma membrane and bind to ligand-free EGF receptors. A negative EGF concentration peak transiently appears at the cell membrane, but it disappears after equilibrium is established. The arrows beside the vertical axis indicate the order of the curves with respect to time.

(C-D) Simulations were started from a state of homogeneous EGF concentration in the extracellular space, and a binding equilibrium between EGF receptor and its ligand at the membrane. Liganded EGFR was added at the left boundary (“plasma membrane”) once at the beginning of the simulation in C, while free EGF was continuously added at the left boundary in D, simulating ligand exocytosis. The concentration of EGF at the right boundary was kept constant by applying a Dirichlet boundary condition. A transient (C) or a permanent, positive ligand concentration peak (D) appears at the plasma membrane. Details of the simulation are described in the section “Simulation of the concentration gradient generated by binding and exocytosis” in the STAR Methods.

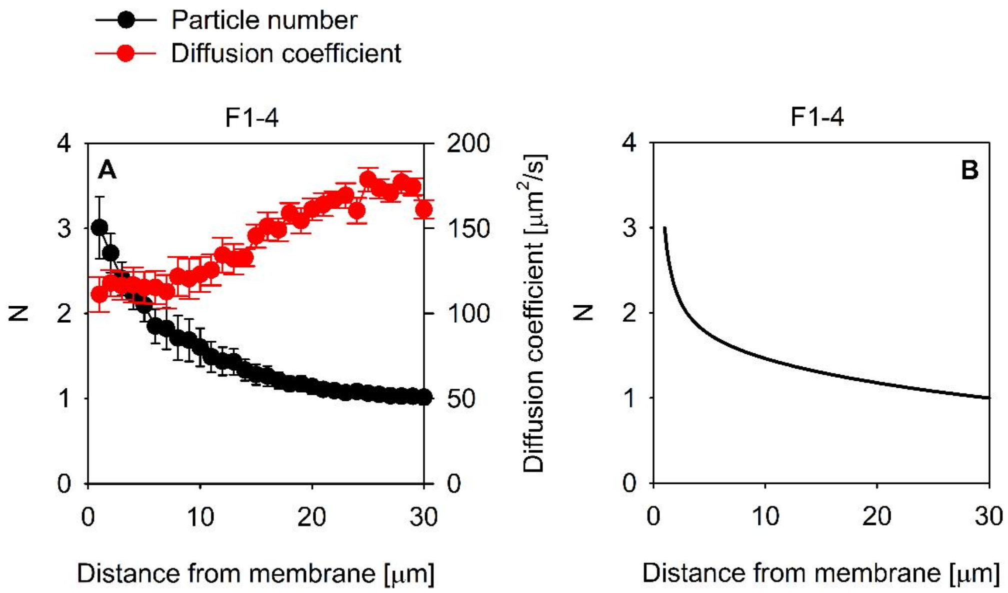


**Supplementary Figure 6. The effect of inhibition of membrane turnover on the concentration profile of EGF.** (**A**) F1-4 cells were pre-treated with a cocktail of 5 μM Latrunculin B, 50 μM para-amino blebbistatin and 50 μM myristoylated dynamin inhibitory peptide at 37°C for 1 hour followed by incubating them with 10 nM TAMRA-EGF. The inhibitors were present throughout the experiment. FCS was used for determining the number of EGF molecules per confocal volume (*N*) and the diffusion coefficient, which are plotted as a function of distance from the membrane. The error bars represent the standard deviation. (**B**) Calculation of the EGF concentration gradient expected due to the non-homogeneous diffusion coefficient of EGF shown in A. The principle of the calculation is identical to that shown in Fig. 2A and described in “Calculation of the local concentration gradient due to locally hindered diffusion” in the STAR Methods. The spatially varying diffusion coefficient of EGF was modeled with a power function followed by solving the Fokker-Planck equation to find the equilibrium concentration profile of EGF, which is remarkably similar to the experimentally observed curve in A.

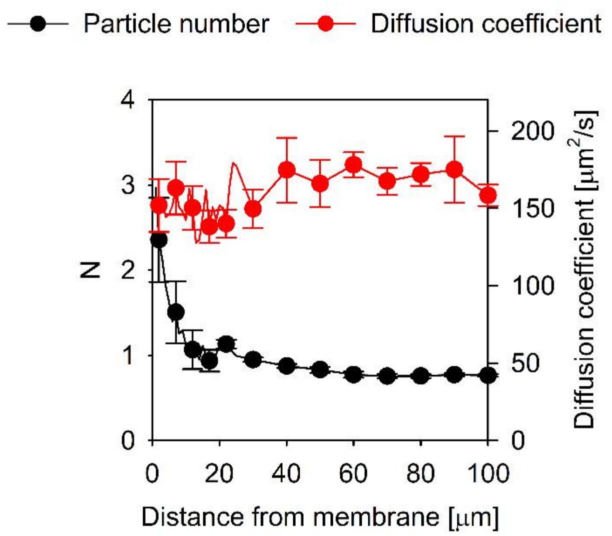


**Supplementary Figure 7. Lack of effect of monensin on the membrane-proximal ligand concentration peak.** F1-4 cells were pre-treated with 10 μM monensin at 37°C for half an hour followed by incubating them with 10 nM TAMRA-EGF. The inhibitor was present throughout the experiment. FCS was used for determining the number of EGF molecules per confocal volume (*N*) and the diffusion coefficient, which are plotted as a function of distance from the membrane. The error bars represent the standard deviation.

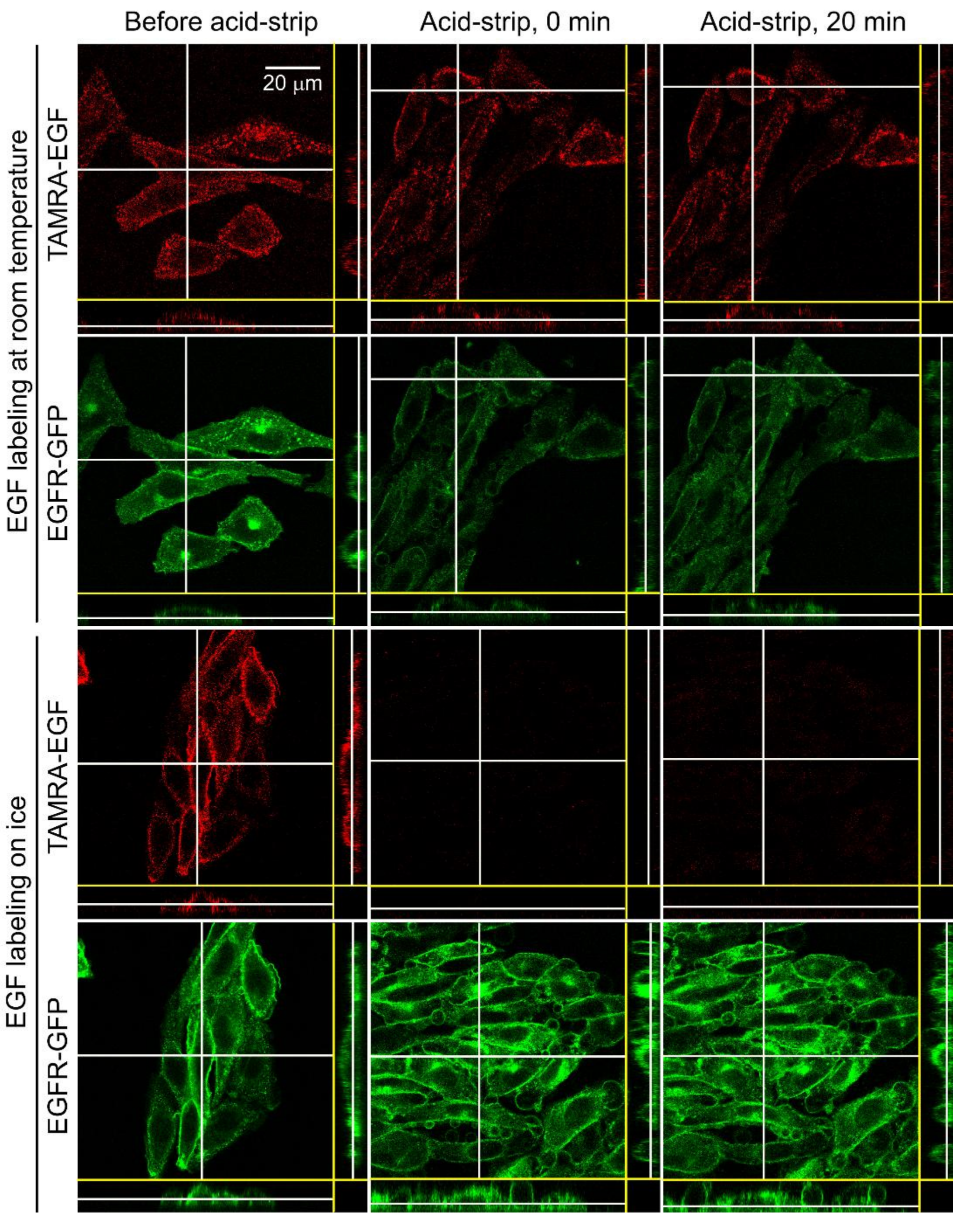


**Supplementary Figure 8. EGF is present in the immediate vicinity of the plasma membrane in an acid wash-resistant compartment.** F1-4 cells expressing EGFR fused to GFP were incubated with 10 nM EGF for 20 min either at room temperature (top panel) or on ice (bottom panel) followed by removing the non-internalized, membrane-bound fraction of EGF by acid stripping (200 mM acetic acid, 500 mM NaCl, pH 2.8, 5 min on ice). Orthogonal views of confocal microscopic stacks recorded before acid washing as well as 0 and 20 min after acid washing are shown in the figure.

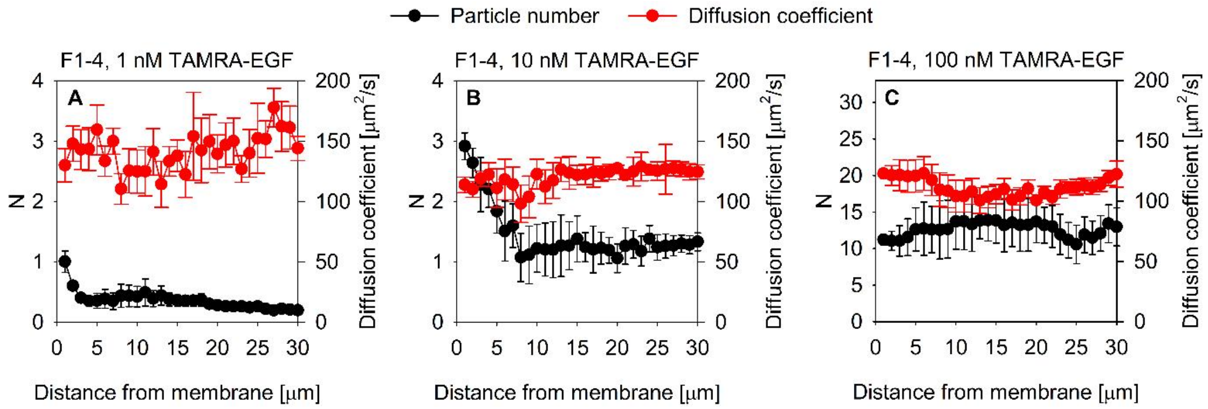


**Supplementary Figure 9. Dependence of the membrane proximal EGF concentration peak on the bulk concentration of EGF.** F1-4 cells were incubated with 1 nM (A), 10 nM (B) or 100 nM (C) TAMRA-EGF, and the number of EGF molecules and the diffusion coefficient of EGF were determined by FCS. The error bars correspond to the standard deviation.

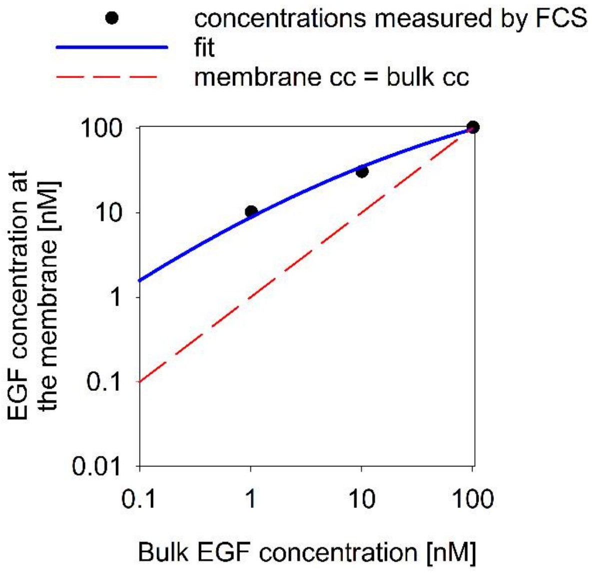


**Supplementary Figure 10. Relationship between the bulk concentration of EGF and the EGF concentration at the membrane.** Concentrations of TAMRA-EGF measured at the membrane (Suppl. Fig. 9) at three different bulk EGF concentrations are shown by the black symbols. The relationship between the bulk ($c_{\text{bulk}}$) and membrane-proximal EGF concentrations ($c_{\text{membrane}}$) was fitted by the following quadratic function:

$$\ln\left(c_{membrane}\right) = -0.0327\,\left[\ln\left(c_{bulk}\right)\right]^2 + 0.6711\,\ln\left(c_{bulk}\right) + 2.172$$

The fitted equation is shown by the blue line. The dashed red line represents the scenario where the bulk and membrane-proximal EGF concentrations are equal.

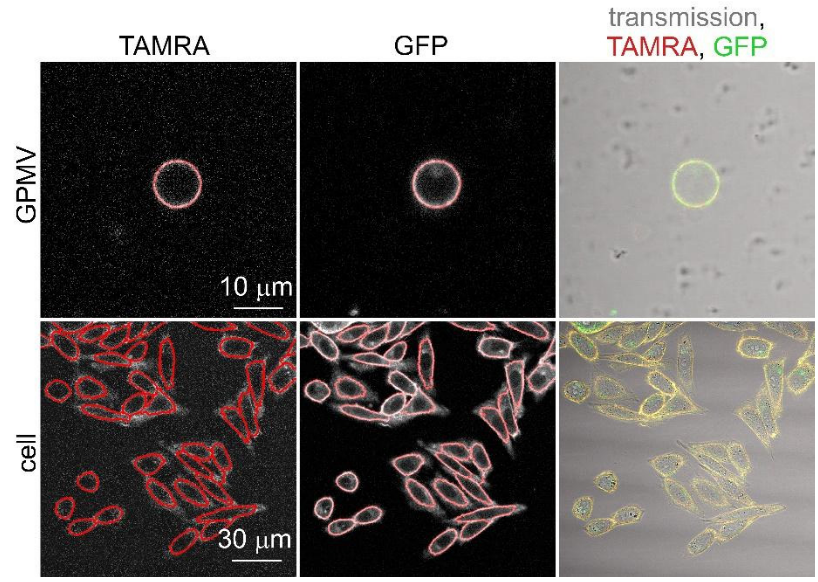


**Supplementary Figure 11. Confocal microscopic images of TAMRA-EGF bound to cells and GPMVs.** GPMVs were generated from F1-4 cells. F1-4 cells were cultured on chambered coverslips, whereas F1-4-derived GPMVs were placed on poly-L-lysine-coated coverslips followed by incubation with 10 nM TAMRA-EGF. Images were recorded in the TAMRA, GFP and transmission channels of a confocal microscope. The plasma membrane was identified by manually-seeded watershed segmentation of the GFP images. The membrane masks are shown in red overlaid on the TAMRA and GFP images. The transmission and both fluorescence images are superimposed in the right column. The scale bars are 10 um and 30 um, as shown in the figure.

## References


1. French, A.R., Tadaki, D.K., Niyogi, S.K., and Lauffenburger, D.A. (1995). Intracellular trafficking of epidermal growth factor family ligands is directly influenced by the pH sensitivity of the receptor/ligand interaction. J Biol Chem *270*, 4334-4340. 10.1074/jbc.270.9.4334.
2. DeWitt, A., Iida, T., Lam, H.Y., Hill, V., Wiley, H.S., and Lauffenburger, D.A. (2002). Affinity regulates spatial range of EGF receptor autocrine ligand binding. Dev Biol *250*, 305-316. 10.1006/dbio.2002.0807.
